# PCSK9 inhibitor use and risk of dementia and neurodegenerative diseases in adults aged 50 Years and older: a global electronic health record study

**DOI:** 10.3389/fphar.2026.1942691

**Published:** 2026-10-29

**Authors:** Shuo-Yan Gau, Shao-Wei Lo, Christine Hsu, Yung-Fang Tu, Yu-Jung Su, Chien-Chin Chen, Meng-Che Wu, Yasue Mitsukura, Hui-Chin Chang

**Affiliations:** 1 Institute of Allergology, Charité-Universitätsmedizin Berlin, Corporate Member of Freie Universität Berlin and Humboldt-Universität zu Berlin, Berlin, Germany; 2 Department of Medical Education, Ditmanson Medical Foundation Chia-Yi Christian Hospital, Chiayi City, Taiwan; 3 Department and Graduate Institute of Business Administration, National Taiwan University, Taipei, Taiwan; 4 Department of Internal Medicine, Cleveland Clinic Foundation, Cleveland, OH, United States; 5 Department of Medicine, Monmouth Medical Center, Robert Wood Johnson/Barnabas Healthcare System, Long Branch, NJ, United States; 6 Department of Biostatistics, Harvard T.H. Chan School of Public Health, Boston, MA, United States; 7 Orthopedics Department, Chi-Mei Medical Center, Tainan, Taiwan; 8 Department of Pathology, Ditmanson Medical Foundation Chia-Yi Christian Hospital, Chiayi, Taiwan; 9 Department of Cosmetic Science, Chia Nan University of Pharmacy and Science, Tainan, Taiwan; 10 Ph.D. Program in Translational Medicine, Rong Hsing Research Center for Translational Medicine, National Chung Hsing University, Taichung, Taiwan; 11 Department of Biotechnology and Bioindustry Sciences, College of Bioscience and Biotechnology, National Cheng Kung University, Tainan, Taiwan; 12 School of Medicine, Chung Shan Medical University, Taichung, Taiwan; 13 Division of Pediatric Gastroenterology, Children’s Medical Center, Taichung Veterans General Hospital, Taichung, Taiwan; 14 Department of Post-Baccalaureate Medicine, College of Medicine, National Chung Hsing University, Taichung, Taiwan; 15 Department of Neuropsychiatry, Faculty of Science and Technology, Keio University School of Medicine, Keio University, Tokyo, Japan; 16 Evidence-based Medicine Center, Chung Shan Medical University Hospital, Taichung, Taiwan; 17 Library, Chung Shan Medical University Hospital, Taichung, Taiwan

**Keywords:** dementia, epidemiology, hyperlipidemia, PCSK9 inhibitor, real-world study

## Abstract

**Background:**

Proprotein convertase subtilisin/kexin type 9 (PCSK9) inhibitors achieve profound lipid lowering, yet their long-term neurocognitive effects remain uncertain. Evidence from randomized trials is limited by short follow-up and lack of dedicated neurodegenerative endpoints.

**Methods:**

We performed a retrospective target trial emulation using the TriNetX Global Collaborative Network. Adults aged ≥50 years with hyperlipidemia initiating PCSK9 inhibitors or comparator lipid-lowering therapies between 2015 and 2024 were included. Active-comparator cohorts were constructed against statins, ezetimibe, and fenofibrate or cholestyramine with mutual exclusion of prior exposure. Propensity score matching at a 1:1 ratio balanced demographics, body mass index, socioeconomic indicators, healthcare utilization, and cardiometabolic comorbidities. Outcomes included incident all-cause dementia, Alzheimer’s disease, vascular dementia, Parkinson’s disease, and other neurodegenerative disorders. Sensitivity analyses incorporated lag periods, varying follow-up durations, alternative matching strategies, and intention-to-treat analyses. Cross-dataset validation was additionally conducted using the US Collaborative Network to assess reproducibility under independent data settings. Hazard ratio along with 95% confidence interval were evaluated in each analysis.

**Results:**

Among 31,604 matched PCSK9 inhibitor–statin pairs, PCSK9 inhibitor use was associated with lower risks of all-cause dementia (HR 0.454; 95% CI 0.394–0.522), Alzheimer’s disease (HR 0.553; 95% CI 0.433–0.705), vascular dementia (HR 0.536; 95% CI 0.402–0.714), Parkinson’s disease (HR 0.686; 95% CI 0.544–0.865), and other neurodegenerative disorders (HR 0.730; 95% CI 0.645–0.825). Results were consistent across comparator therapies, cross-dataset validation and sensitivity analyses.

**Conclusion:**

PCSK9 inhibitor use was associated with lower observed risks of neurodegenerative outcomes across multiple analyses and datasets. These findings support a potential association but do not establish causality and require confirmation in prospective trials.

## Introduction

Lipid-lowering therapy is a cornerstone of atherosclerotic cardiovascular disease (ASCVD) prevention, with compelling evidence demonstrating substantial reductions in myocardial infarction, stroke, and cardiovascular mortality (Chou et al., 2022; Newman et al., 2020). Beyond macrovascular outcomes, accumulating epidemiologic and mechanistic data implicate dyslipidemia and ASCVD in the pathogenesis of cognitive decline and dementia. Midlife hypercholesterolemia has been associated with more than a twofold increased risk of Alzheimer’s disease (AD) compared with normocholesterolemia, highlighting a potential window during which lipid exposure may exert long-term effects on brain health (Anstey et al., 2017). These associations are thought to be mediated through cerebrovascular injury, chronic neuroinflammation, and perturbations in amyloid and tau metabolism, raising the possibility that lipid-lowering interventions, particularly when initiated earlier in the disease course, could modify future dementia risk (Goldstein et al., 2023; Wu et al., 2022).

Despite this strong biological plausibility, randomized clinical trials (RCTs) of statins, the foundation of lipid-lowering therapy, have not demonstrated a clear benefit in preventing cognitive decline or dementia (Collins et al., 2016; Zhou et al., 2021). Attention has therefore shifted toward non-statin lipid-lowering therapies, most notably proprotein convertase subtilisin/kexin type 9 inhibitors, which achieve profound reductions in low-density lipoprotein cholesterol (LDL-C). Large RCTs have established robust cardiovascular benefits of PCSK9 inhibitors, accompanied by improvements in endothelial and microvascular function and reductions in systemic inflammation (Ray et al., 2019; Imran et al., 2023). Importantly, PCSK9 is expressed in the central nervous system and is upregulated in AD brains (Picard et al., 2019; Bell et al., 2023a). However, human evidence regarding the neurocognitive consequences of PCSK9 inhibition remains incomplete. Clinical trials and meta-analyses evaluating neurocognitive dysfunction primarily as an adverse safety outcome have not identified an increased risk associated with PCSK9 inhibitors use (Giugliano et al., 2017; Hirsh et al., 2021). Nevertheless, these trials were not designed or powered to assess incident neurodegenerative diseases, had relatively short follow-up, and enrolled highly selected populations (Giugliano et al., 2017; Hirsh et al., 2021). Consequently, the effects of PCSK9 inhibitors on the risk of neurodegenerative conditions remain uncertain.

Recent Mendelian randomization evidence is mixed rather than uniformly neutral. A Danish study found no evidence that genetically lower LDL-C through PCSK9 and HMGCR increased the risk of Alzheimer’s disease, vascular dementia, all-cause dementia, or Parkinson’s disease, although a possible reduction in Alzheimer’s disease could not be excluded (Benn et al., 2017). In a Mendelian randomization meta-analysis of 1,091,775 individuals, genetically proxied PCSK9-mediated non-HDL-C lowering showed a neutral association with dementia risk (OR 0.97, 95% CI 0.70–1.35 per 1 mmol/L lower non-HDL-C), whereas instruments for HMGCR, NPC1L1, and CETP were each associated with significantly lower dementia risk (Nordestgaard et al., 2025). A recent drug-target Mendelian randomization study of vascular dementia found little evidence supporting most lipid-lowering targets after correction for multiple testing (Taylor-Bateman et al., 2026). To address these gaps, we utilized a large-scale, global federated health research network to conduct target emulated trials comparing the incidence of neurodegenerative disorders among new users of PCSK9 inhibitors versus users of statins and other lipid-lowering therapies, including ezetimibe, fenofibrate, and cholestyramine. Outcomes of interest included all-cause dementia, Alzheimer’s disease, vascular dementia, Parkinson’s disease, and other neurodegenerative conditions.

## Materials and methods

### Data origin and analytical framework

This study utilized real-world clinical data sourced from the TriNetX research network, a large, international federated system that consolidates routinely collected healthcare information from over 130 contributing medical institutions. The platform provides access to de-identified electronic health records, including diagnostic classifications, laboratory measurements, procedural records, prescription data, behavioral indicators, and selected markers of socioeconomic context. For the present analysis, we utilized data from the Global Collaborative Network, a TriNetX component encompassing more than 150 healthcare organizations located globally.

All analyses were conducted using anonymized observational data, with no direct interaction with patients. The overall study design was structured to emulate the conditions of a randomized controlled trial through a target trial emulation strategy applied to real-world evidence. Detailed operational definitions, inclusion rules, and analytic coding specifications are provided in Supplementary Tables S1, S2.

### Study period and eligibility criteria

The analytic timeframe spanned from 1 January 2015, to 31 December 2024. Cohort entry was defined by the first documented prescription of either a PCSK9 inhibitors or a comparator lipid-lowering medication, including statins, ezetimibe, fenofibrates, or cholestyramine. Baseline covariates were ascertained using all available clinical data recorded up to 1 day prior to the index prescription date. This period was selected because PCSK9 inhibitors entered routine clinical use in 2015, allowing contemporary exposure ascertainment while providing the longest feasible observation window through the latest complete year of data.

Participants were required to be 50 years of age or older, carry a documented diagnosis of hyperlipidemia, and represent new users of the study medications. Individuals with any preexisting diagnosis of dementia, Alzheimer’s disease, Parkinson’s disease, or other neurodegenerative disorders were excluded to ensure incident outcome assessment. All diagnoses and medication exposures were identified using standardized administrative coding systems, as outlined in Supplementary Table S2.

### Cohort definition and comparative design

An active-comparator approach was implemented through two separately emulated target trials, with cohort derivation summarized in Figure 1.

- PCSK9 inhibitors versus statins:

**FIGURE 1:**
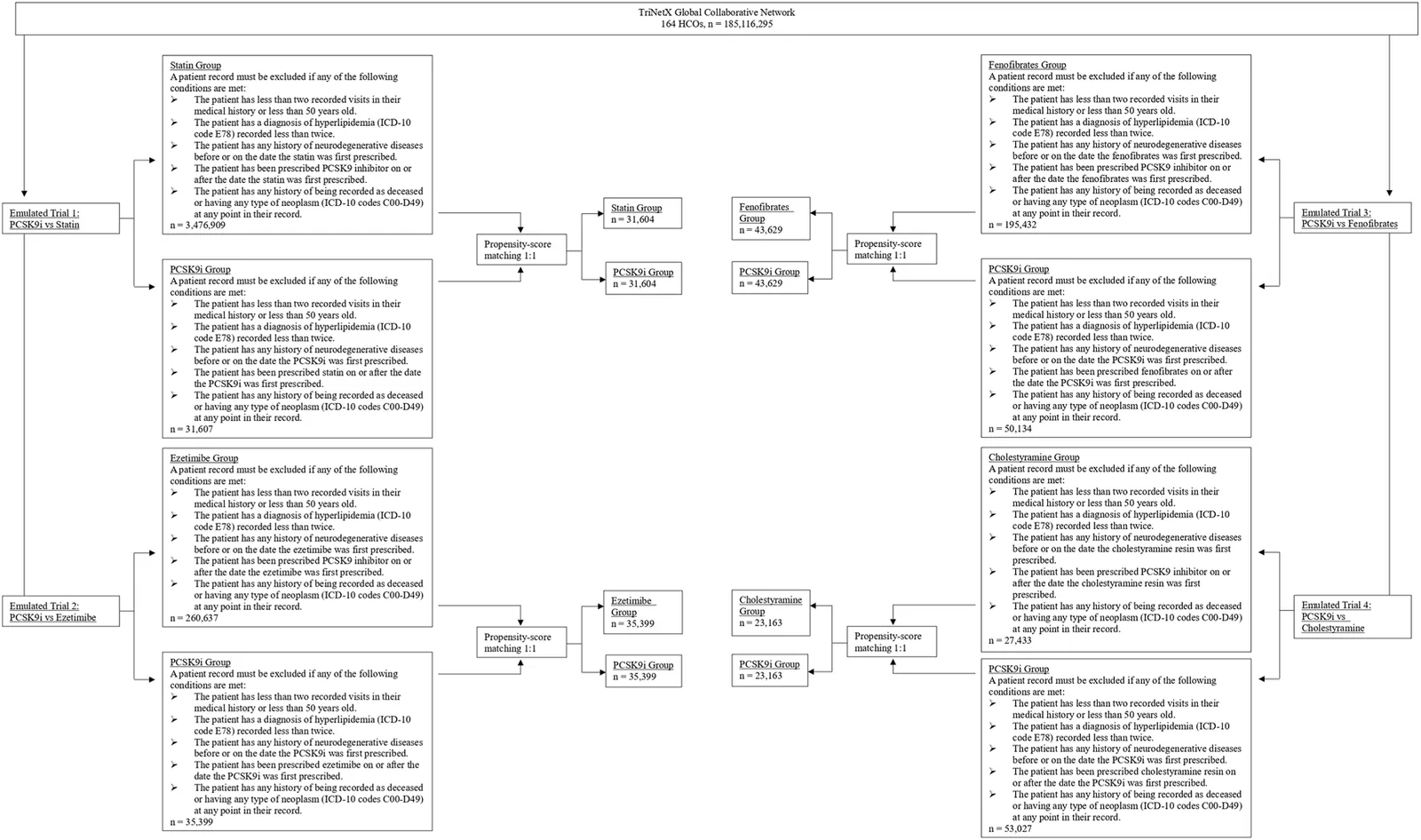
Patient selection flowchart.

Patients were categorized into mutually exclusive exposure groups. The PCSK9 inhibitor cohort consisted of individuals with no recorded statin use at any point in their medical history, whereas the statin cohort included patients with no evidence of PCSK9 inhibitor exposure. This strict mutual-exclusion definition was retained for the primary analysis to preserve a clearly separated active-comparator contrast; an additional sensitivity analysis used a statin-unrestricted PCSK9 inhibitor cohort to evaluate the influence of this design choice.

- PCSK9 inhibitors versus ezetimibe/fenofibrates/cholestyramine:

In the second comparison, one group comprised patients treated with PCSK9 inhibitors who had never received ezetimibe, fenofibrates, or cholestyramine. The comparator group included patients prescribed ezetimibe, fenofibrates, or cholestyramine with no documented history of PCSK9 inhibitor use.

### Outcome definitions and follow-up

The primary endpoint was the first recorded diagnosis of all-cause dementia. Secondary outcomes were vascular dementia, Alzheimer’s disease, Parkinson’s disease, and other degenerative diseases of the nervous system. The all-cause dementia definition included specified and unspecified dementia diagnoses. Dementia subtype diagnoses were not mutually exclusive; therefore, the proportion of all-cause dementia represented by Alzheimer’s disease or vascular dementia could not be calculated reliably from the aggregate platform output. Follow-up began on the date of treatment initiation and continued until the earliest occurrence of an outcome event or the end of the prespecified observation window.

### Confounding control and matching procedures

To reduce confounding and improve comparability between treatment groups, propensity score matching was employed to balance baseline characteristics associated with treatment assignment. Matching models incorporated demographic factors (age at index date, sex, and race), body mass index, socioeconomic and behavioral indicators (including socioeconomic or psychosocial health hazards, family history of mental or behavioral disorders [Z81.8], and substance-related mental or behavioral conditions), healthcare utilization metrics (outpatient and inpatient encounters), and a predefined set of comorbid conditions. These comorbidities included essential hypertension, diabetes mellitus, chronic ischemic heart disease, prior cerebral infarction, chronic kidney disease, and vitamin D deficiency.

### Sensitivity and stratified analyses

The primary restricted-exposure analysis excluded individuals with documented crossover to the alternative therapy during follow-up. Because the TriNetX interface does not support time-varying censoring with inverse-probability adjustment at the exact date of switching, an as-started intention-to-treat sensitivity analysis retained patients in their initial exposure groups regardless of subsequent treatment changes. Additional sensitivity analyses incorporated exposure lag periods of 6 months, 1 year, and 2 years, along with follow-up windows of one to 4 years (Supplementary Table S3). Cross-dataset validation was performed using the US Collaborative Network. Subgroup analyses were conducted by age category, sex, race, and recorded baseline laboratory categories. To assess the strict primary exposure definition, we also performed a statin-unrestricted sensitivity analysis in which PCSK9 inhibitor users were not excluded for statin use before, on, or after the index date. Its expanded propensity-score model included clinically defined lipid categories and baseline concomitant medications.

### Statistical analysis

All statistical analyses were executed within the TriNetX Analytics platform. We implemented a 1:1 nearest-neighbor matching algorithm based on propensity scores. Hazard ratios accompanied by 95% confidence intervals were respectively evaluated in each analysis. To refine the accuracy of these matches, the maximum allowable difference was set to a caliper width of 0.1 of the logit propensity score’s standard deviation. Post-matching balance was assessed using standardized mean differences, with values greater than 0.1 indicating meaningful residual imbalance. Laboratory values were not imputed. Continuous laboratory summaries used available observations, whereas laboratory-stratified analyses required a recorded value meeting the prespecified category; patients without a qualifying laboratory record remained in the overall cohort but did not contribute to that laboratory subgroup. A Schoenfeld residual-based assessment was not performed. Formal proportional-hazards diagnostics beyond the functionality available in the aggregate TriNetX output were not available for independent verification. We therefore assessed the temporal stability of the estimates across prespecified follow-up windows, while acknowledging that these analyses do not substitute for a full proportional-hazards assessment. Sensitivity analyses across restricted follow-up windows were used to examine the stability of estimates over time. Formal competing-risk regression was also unavailable. We therefore repeated the restricted-exposure and as-started analyses without excluding patients with a recorded death, while recognizing that this does not replace a cause-specific or Fine-Gray competing-risk analysis.

## Results

### Study population and baseline characteristics

In the primary emulated trial comparing PCSK9 inhibitors with statins, we identified 31,607 new users of PCSK9 inhibitor and 3,476,909 new users of statins. Before propensity-score matching, patients initiating PCSK9 inhibitor were older (mean age 64.5 ± 10.7 vs. 61.0 ± 13.3 years) and exhibited a higher cardiovascular risk profile, including a greater prevalence of chronic ischemic heart disease (52.0% vs. 14.3%) and essential hypertension (67.4% vs. 47.5%) (Table 1). After 1:1 propensity-score matching, 31,604 patients were retained in each group, with adequate balance for the covariates included in the primary matching model. In the matched cohorts, the mean age was 64.4 years, 51% of participants were female, and the racial distribution was 81% White and 8% Black or African American. Similar balance in the prespecified matching covariates was achieved in the secondary emulated trials comparing PCSK9 inhibitor with ezetimibe (35,399 matched pairs), fenofibrate (43,629 matched pairs), and cholestyramine (23,163 matched pairs) (Supplementary Tables S4–S6).

**TABLE 1:** Baseline characteristics of study population (before and after propensity score matching) in emulated trial 1 (PCSK9 inhibitor *versus* Statin).

​	Before matching			After matching^a^		
	PCSK9 inhibitors group (n = 31,607)	Statin group (n = 3,476,909)	SMD	PCSK9 inhibitors group (n = 31,604)	Statin group (n = 31,604)	SMD
Age at index						
Mean ± SD	64.5 ± 10.7	61.0 ± 13.3	0.30	64.4 ± 11.0	64.4 ± 10.9	0.01
Sex						
Male	15463 (48.9)	1881156 (54.1)	0.10	15463 (48.9)	15454 (48.9)	0.00
Female	16101 (50.9)	1593225 (45.8)	0.10	16101 (50.9)	16115 (51.0)	0.00
Unknown Gender	43 (<0.1)	2528 (<0.1)	0.02	40 (<0.1)	35 (<0.1)	0.00
Race, n (%)						
White	25314 (80.1)	2230886 (64.2)	0.36	25311 (80.1)	25448 (80.5)	0.01
Black or African American	2614 (8.3)	455392 (13.1)	0.16	2614 (8.3)	2660 (8.4)	0.01
Asian	789 (2.5)	167301 (4.8)	0.12	789 (2.5)	764 (2.4)	0.01
American Indian or Alaska Native	130 (0.4)	14061 (0.4)	0.00	130 (0.4)	98 (0.3)	0.02
Native Hawaiian or other Pacific Islander	67 (0.2)	17529 (0.5)	0.05	67 (0.2)	70 (0.2)	0.00
Other race	901 (2.9)	121386 (3.5)	0.04	901 (2.9)	863 (2.7)	0.01
Unknown race	1792 (5.7)	470354 (13.5)	0.27	1792 (5.7)	1701 (5.4)	0.01
Baseline lipid profile, n (%)						
Triglyceride in Serum, Plasma or blood						
≧ 200 mg/dL	9087 (28.8)	512131 (14.7)	0.34	9086 (28.8)	5321 (16.8)	0.29
Cholesterol in HDL in Serum or Plasma	​	​	​	​	​	​
≤40 mg/dL	8868 (28.1)	601462 (17.3)	0.26	8867 (28.1)	6846 (21.7)	0.15
Cholesterol in LDL in Serum or Plasma	​	​	​	​	​	​
≧ 130 mg/dL	14111 (44.6)	789058 (22.7)	0.48	14110 (44.6)	7396 (23.4)	0.46
Cholesterol in Serum or Plasma	​	​	​	​	​	​
≧ 200 mg/dL	15295 (48.4)	920348 (26.5)	0.47	15293 (48.4)	8799 (27.8)	0.43
Other lab data profile, n (%)						
Urate level in Serum or Plasma	​	​	​	​	​	​
≧ 7.5 mg/dL	837 (2.6)	65881 (1.9)	0.05	837 (2.6)	674 (2.1)	0.03
HbA1c	​	​	​	​	​	​
≧ 5.7%	11107 (35.1)	875563 (25.2)	0.22	11104 (35.1)	9310 (29.5)	0.12
BMI, n (%)						
≧ 25 (kg/m^2^)	19408 (61.4)	1446901 (41.6)	0.40	19406 (61.4)	19326 (61.2)	0.01
Medical utilization status						
Visit: Ambulatory	26562 (84.0)	2474847 (71.2)	0.31	26562 (84.0)	26705 (84.5)	0.01
Visit: Inpatient encounter	9057 (28.7)	731897 (21.1)	0.18	9057 (28.7)	9185 (29.1)	0.01
Socioeconomic status						
Persons with potential health hazards related to socioeconomic and psychosocial circumstances	544 (1.7)	50442 (1.5)	0.02	544 (1.7)	448 (1.4)	0.02
Family history						
Family history of other mental and behavioral disorders	67 (0.2)	5684 (<0.1)	0.01	67 (0.2)	40 (0.1)	0.02
Family history of other endocrine, nutritional and metabolic diseases	209 (0.7)	10735 (0.3)	0.05	209 (0.7)	119 (0.4)	0.04
Lifestyle						
Mental and behavioral disorders due to psychoactive substance use	4483 (14.2)	412899 (11.9)	0.07	4482 (14.2)	4468 (14.1)	0.00
Comorbidities						
Essential hypertension	21288 (67.4)	1649989 (47.5)	0.41	21285 (67.4)	21232 (67.2)	0.00
Chronic ischemic heart disease	16448 (52.0)	498689 (14.3)	0.87	16445 (52.0)	16485 (52.2)	0.00
Diabetes mellitus	9303 (29.4)	899931 (25.9)	0.08	9302 (29.4)	9288 (29.4)	0.00
Vitamin D deficiency	5560 (17.6)	370899 (10.7)	0.20	5558 (17.6)	5354 (16.9)	0.02
Chronic kidney disease	3334 (10.6)	237077 (6.8)	0.13	3334 (10.6)	3236 (10.2)	0.01
Diseases of liver	2916 (9.2)	167288 (4.8)	0.17	2915 (9.2)	1809 (5.7)	0.13
Cerebral infarction	1862 (5.9)	126559 (3.6)	0.11	1862 (5.9)	1802 (5.7)	0.01
Comedications						
Vasoprotectives	19068 (60.3)	1104481 (31.8)	0.60	19066 (60.3)	14503 (45.9)	0.29
Beta blockers	16306 (51.6)	700483 (20.2)	0.69	16304 (51.6)	10687 (33.8)	0.37
Agents acting on the renin-angiotensin system	15649 (49.5)	829255 (23.9)	0.55	15648 (49.5)	10051 (31.8)	0.37
Aspirin	15066 (47.7)	521477 (15.0)	0.75	15064 (47.7)	8489 (26.9)	0.44
Corticosteroids	14590 (46.2)	908966 (26.1)	0.43	14588 (46.2)	10564 (33.4)	0.26
Diuretics	11781 (37.3)	616762 (17.7)	0.45	11779 (37.3)	7836 (24.8)	0.27
Calcium channel blockers	11444 (36.2)	508078 (14.6)	0.51	11442 (36.2)	6595 (20.9)	0.34
Vitamins	10250 (32.4)	440197 (12.7)	0.49	10248 (32.4)	5603 (17.7)	0.34
Immunosuppressants	1507 (4.8)	59777 (1.7)	0.17	1507 (4.8)	714 (2.3)	0.14
Niacin	1053 (3.3)	26688 (0.8)	0.18	1052 (3.3)	418 (1.3)	0.13

PCSK9 inhibitor, Proprotein Convertase Subtilisin/Kexin Type 9 Inhibitor; HDL, High-Density Lipoprotein; LDL, Low-Density Lipoprotein; BMI, body mass index; SD, standardized difference; SMD, standardized mean difference. A standardized mean difference (SMD) exceeding 0.1 was used to define a clinically relevant difference between comparison groups. In the TriNetX analytics platform, to maintain patient deidentification, incident case counts of 10 or fewer are censored and displayed as 10. Propensity score matching was implemented while controlling for baseline demographic variables (index age, sex, and race), body mass index, socioeconomic and lifestyle characteristics (socioeconomic or psychosocial–related health hazards, Family history of other mental and behavioral disorders (Z81.8), and mental or behavioral disorders associated with psychoactive substance use), healthcare utilization patterns (ambulatory and inpatient encounters), and a set of prespecified comorbidities, including essential hypertension, diabetes mellitus chronic ischemic heart disease, cerebral infarction, chronic kidney disease, and vitamin D deficiency.

### Risk of dementia and neurodegenerative disorders

PCSK9 inhibitor use was consistently associated with a significantly reduced risk of neurodegenerative outcomes compared to statins and all other lipid-lowering agents (Figure 2). In the primary as-treated analysis, PCSK9 inhibitor use was associated with a 55% lower risk of all-cause dementia (HR: 0.454; 95% CI: 0.394–0.522), a 46% lower risk of vascular dementia (HR: 0.536; 95% CI: 0.402–0.714), a 45% lower risk of Alzheimer’s disease (HR: 0.553; 95% CI: 0.433–0.705). The risk of Parkinson’s disease (HR: 0.686; 95% CI: 0.544–0.865) and other degenerative neurologic diseases (HR: 0.730; 95% CI: 0.645–0.825) were also significantly reduced.

**FIGURE 2:**
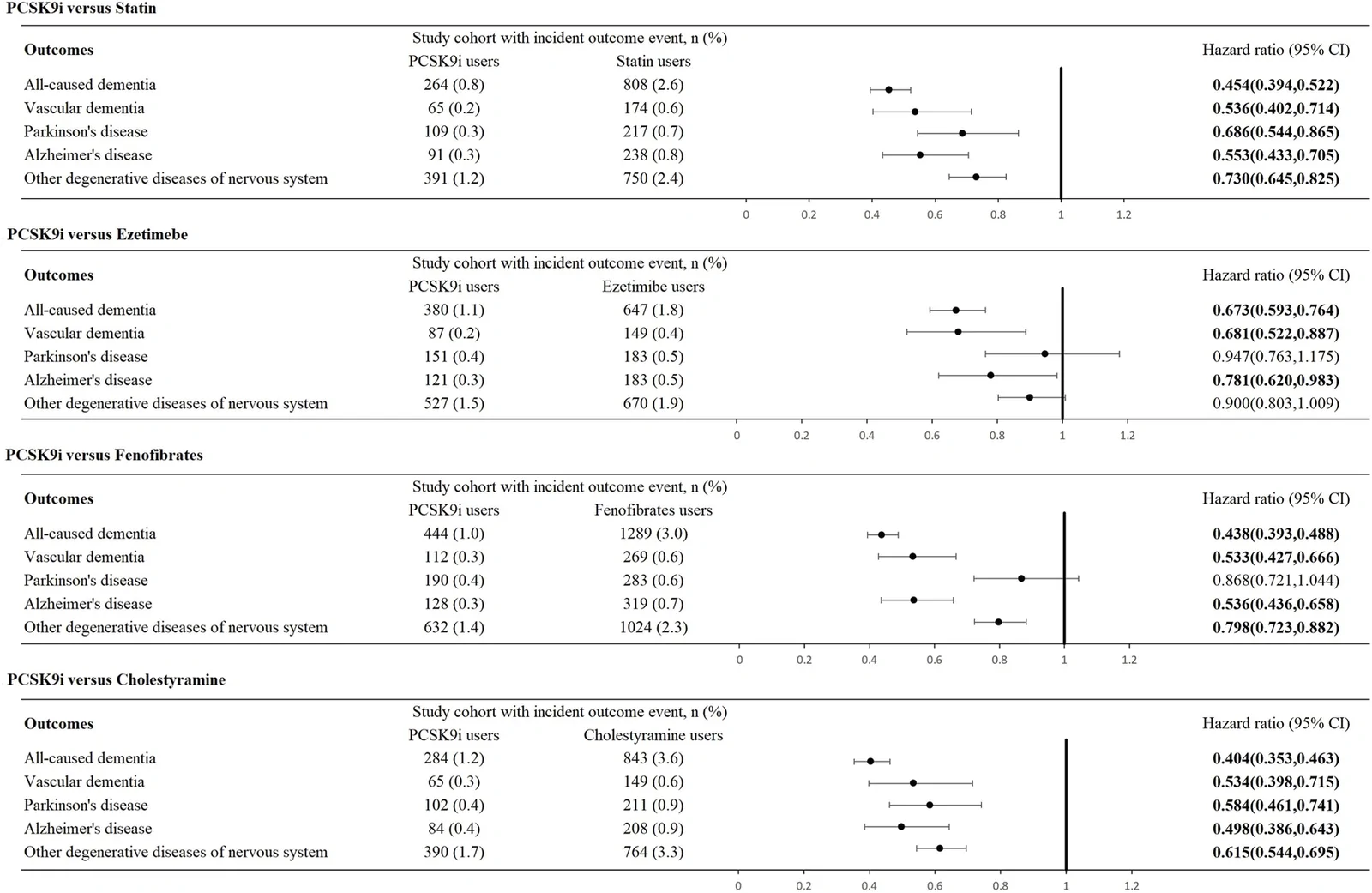
Risk of all-caused dementia, Vascular dementia, Parkinson’s disease, Alzheimer’s disease and other neurodegenerative disorders Comparing the PCSK9 inhibitor Group, Statin Group, Ezetimibe group, Fenofibrates group and Cholestyramine group. In each analysis, lagging period was set as 1 month after prescription and monitoring period was set as 5 years after index date.

In other emulated trials comparing non-statin lipid-lowering therapies, PCSK9 inhibitor use was consistently associated with a lower risk of dementia outcomes (Figure 2). Compared with ezetimibe, fenofibrate, and cholestyramine, PCSK9 inhibitor therapy was associated with relative risk reductions of approximately 30%–60% for all-cause dementia, with HRs ranging from 0.404 (vs. cholestyramine) to 0.673 (vs. ezetimibe). Associations for vascular dementia and Alzheimer’s disease followed a similar pattern. For other degenerative neurologic diseases, comparative estimates were generally concordant with the dementia findings. In contrast, results for Parkinson’s disease were more variable. PCSK9 inhibitor use was associated with a significantly lower risk compared with statins and cholestyramine (HR: 0.584; 95% CI: 0.461–0.741), whereas comparisons with ezetimibe and fenofibrate were not statistically significant. No meaningful differences were observed between monoclonal antibody (mAb)–based PCSK9 inhibitor and small interfering RNA (siRNA)–based PCSK9 inhibitor across all neurodegenerative diseases (Supplementary Table S7). In the result of cross-dataset validation based on the US Collaborative Network, applying the same analytical criteria as the primary analysis (up to 5 years of follow-up and a 1-month washout period), PCSK9 inhibitor use was consistently associated with a lower risk of neurodegenerative outcomes compared with multiple lipid-lowering drug cohorts. Compared with statin, ezetimibe, fenofibrate, and cholestyramine users, PCSK9 inhibitor users showed significantly reduced risks of all-cause dementia (HRs ranging from 0.42 to 0.71), vascular dementia (HRs 0.49–0.72), and Alzheimer’s disease (HRs 0.43–0.71). A lower risk of Parkinson’s disease was also observed in most comparisons, although the association was not significant when compared with ezetimibe users (HR 0.99, 95% CI 0.77–1.27) (Supplementary Table S8).

### Sensitivity analyses

Prespecified sensitivity analyses demonstrated that the observed associations between PCSK9 inhibitor use and reduced dementia risk were highly robust across alternative exposure definitions and follow-up requirements (Supplementary Tables S9–S15; Figures 3–7). Extending the wash-out period from 6 months to 2 years and requiring progressively longer minimum follow-up durations (≥1 to ≥4 years) yielded consistent effect estimates. For all-cause dementia, HRs remained tightly clustered between 0.34 and 0.48 across all specifications (Figure 3), while estimates for vascular dementia (Figure 4), Alzheimer’s disease (Figure 5), and other degenerative neurologic conditions remained within a narrow and consistently protective range (Figure 7). Alternative matching algorithms and intention-to-treat analyses further supported these findings (Supplementary Tables S16–S18; Figures 3–7). In the ITT analysis, PCSK9 inhibitor use remained significantly associated with a lower risk of all-cause dementia (HR: 0.528; 95% CI: 0.479–0.582; Figure 3), vascular dementia (HR: 0.618; 95% CI: 0.506–0.754; Figure 4), Alzheimer’s disease (HR: 0.630; 95% CI: 0.525–0.756; Figure 5), and other degenerative neurologic diseases (HR: 0.833; 95% CI: 0.462–0.910; Figure 7) while comparing with statin users. In contrast, although point estimates for Parkinson’s disease generally favored PCSK9 inhibitor use, confidence intervals frequently crossed unity, and effect sizes varied more substantially than those observed for dementia outcomes (Figure 6). Similar findings were observed in the comparison with ezetimibe, fenofibrates and cholestyramine (Supplementary Table S19).

**FIGURE 3:**
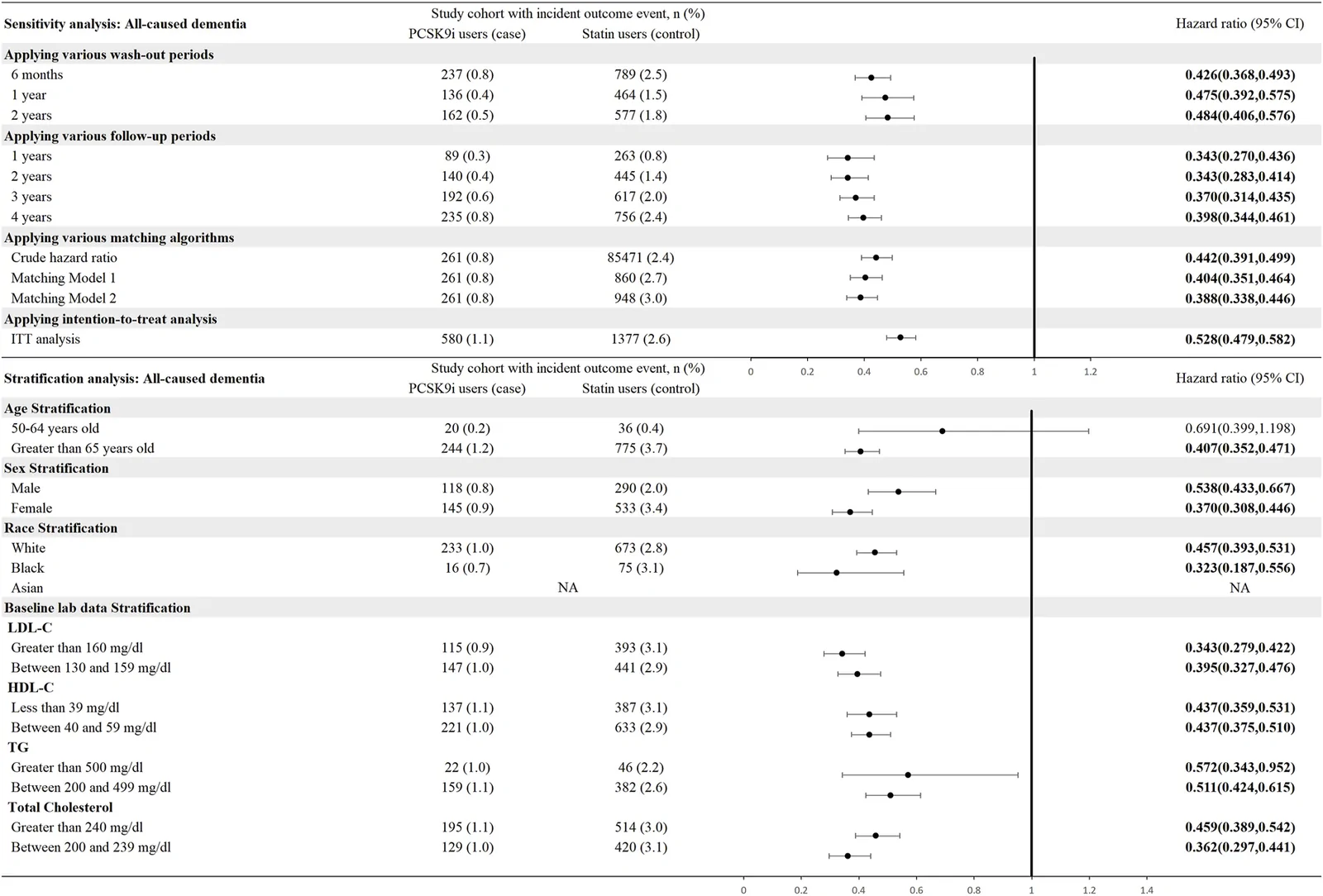
Risk of all-caused dementia in different sensitivity and stratification models comparing the PCSK9 inhibitor Group and Statin Group.

**FIGURE 4:**
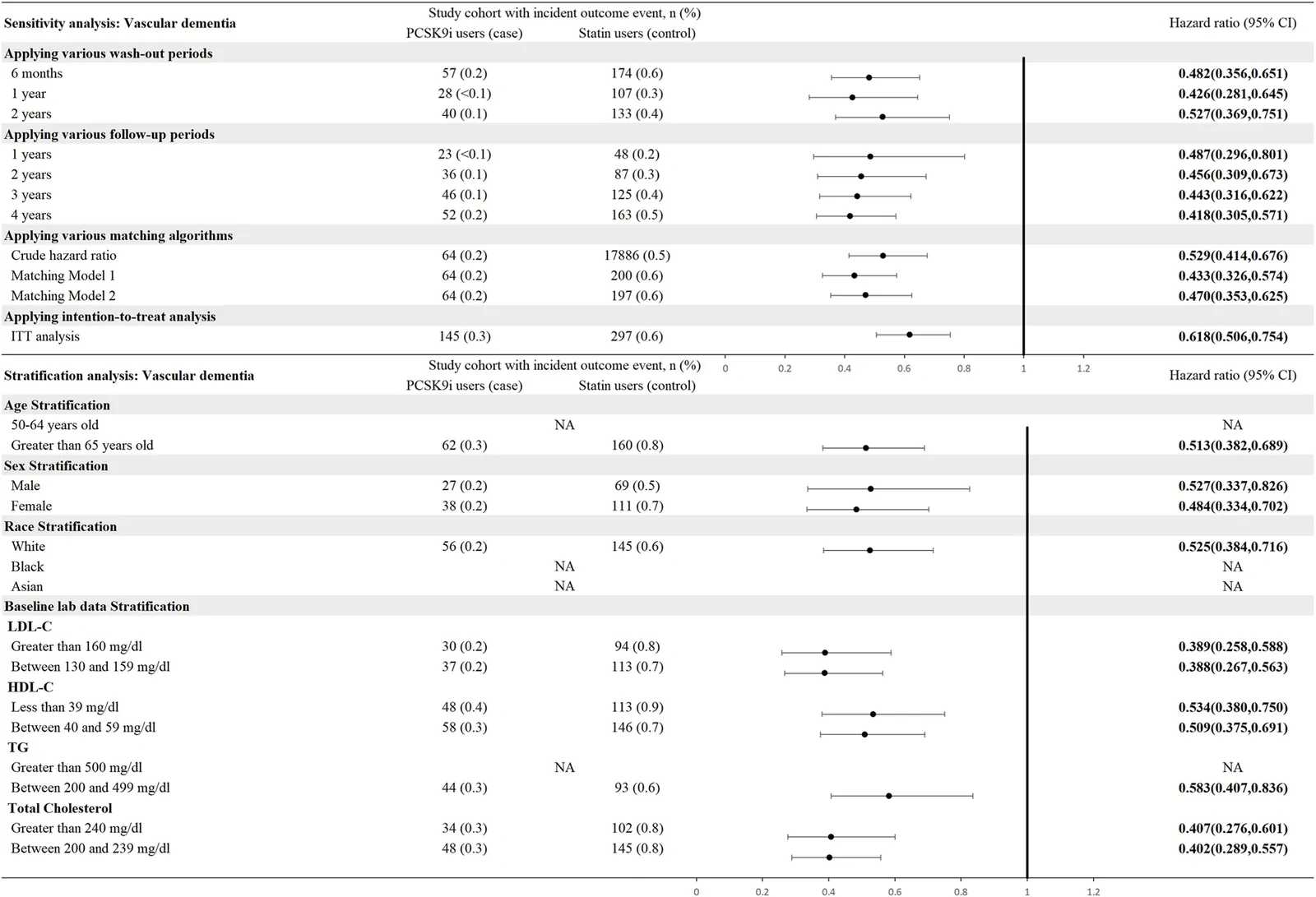
Risk of vascular dementia in different sensitivity and stratification models comparing the PCSK9 inhibitor Group and Statin Group.

**FIGURE 5:**
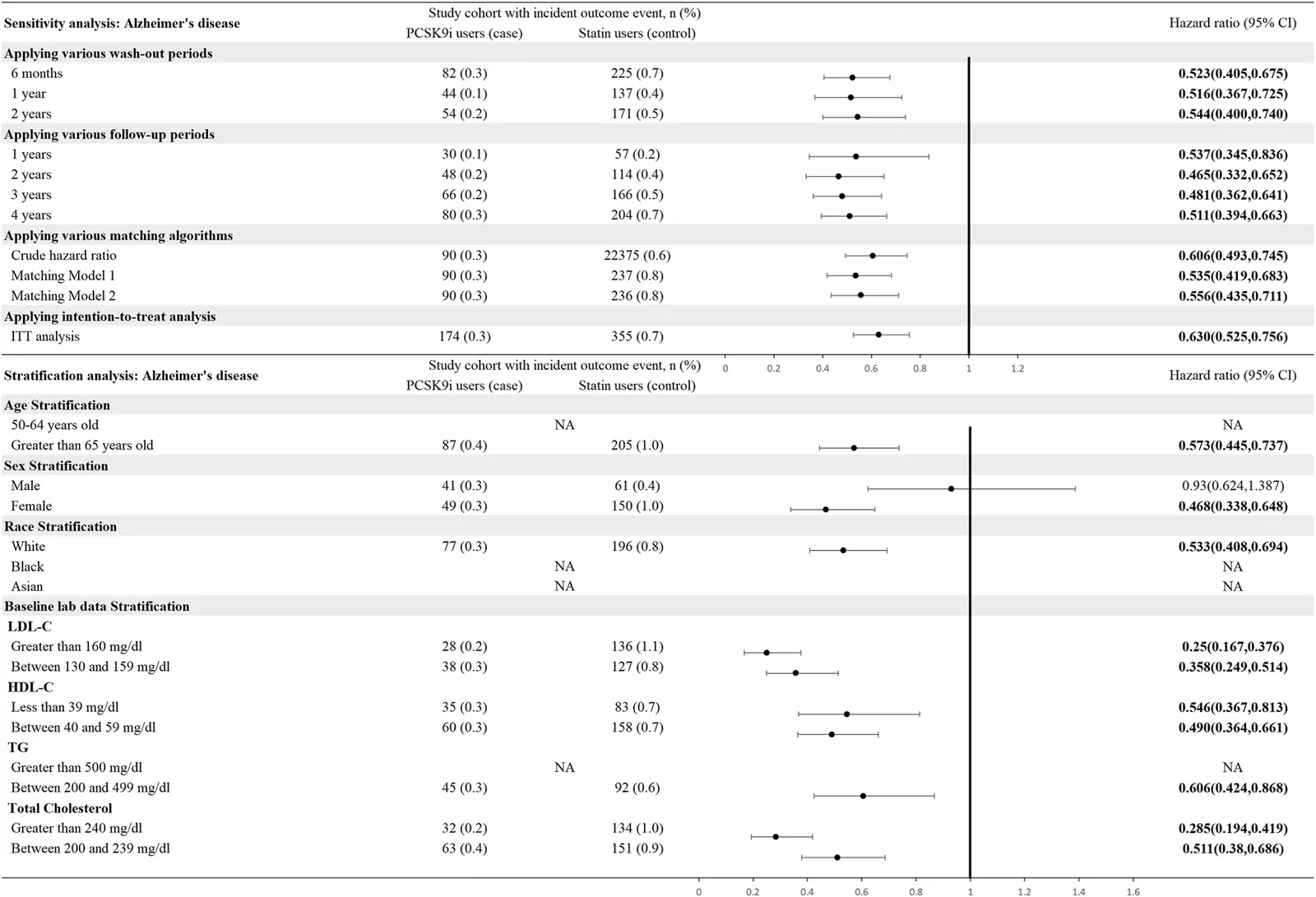
Risk of Alzheimer’s disease in different sensitivity and stratification models comparing the PCSK9 inhibitor group and statin group.

**FIGURE 6:**
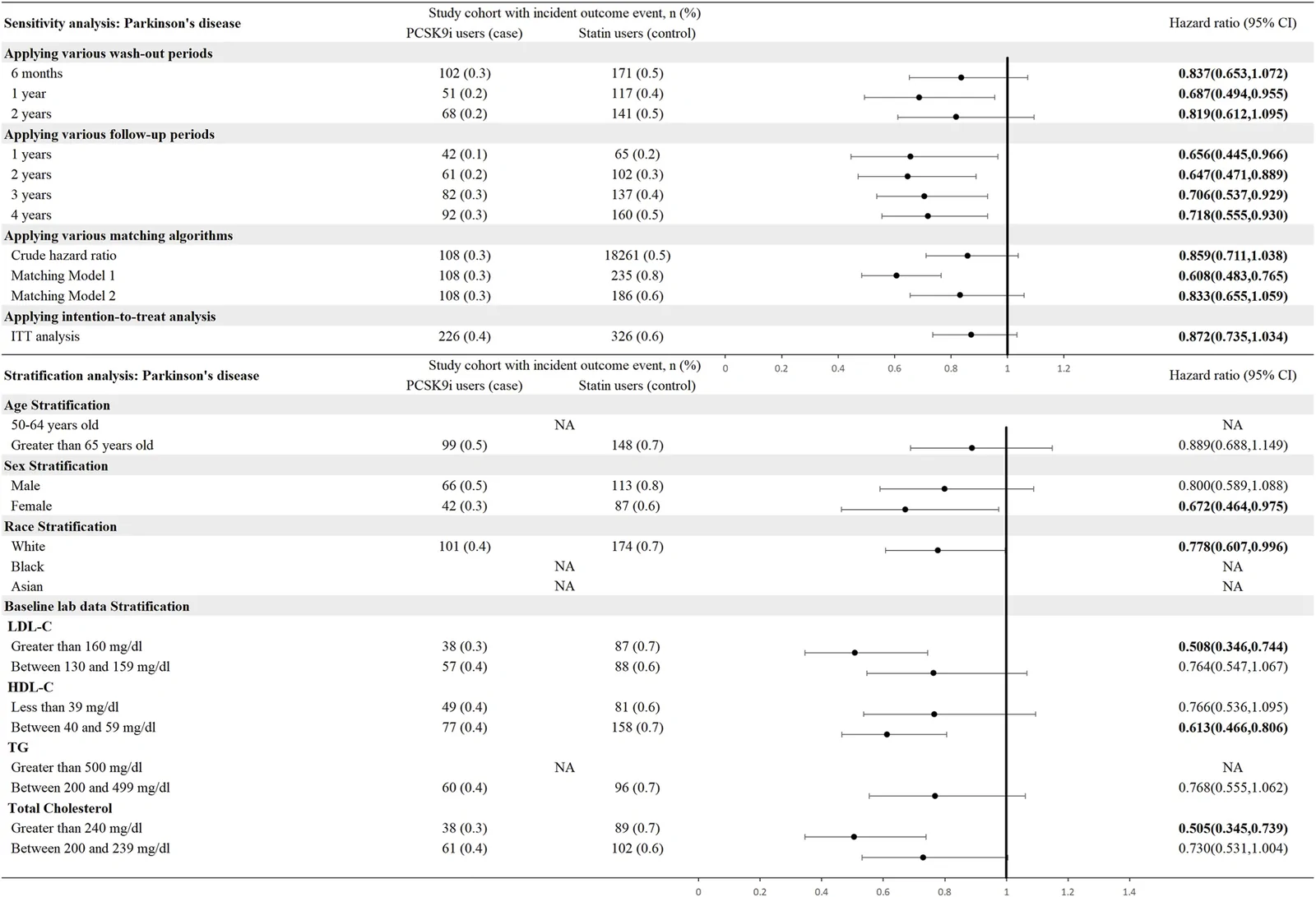
Risk of Parkinson’s disease in different sensitivity and stratification models comparing the PCSK9 inhibitor group and statin group.

**FIGURE 7:**
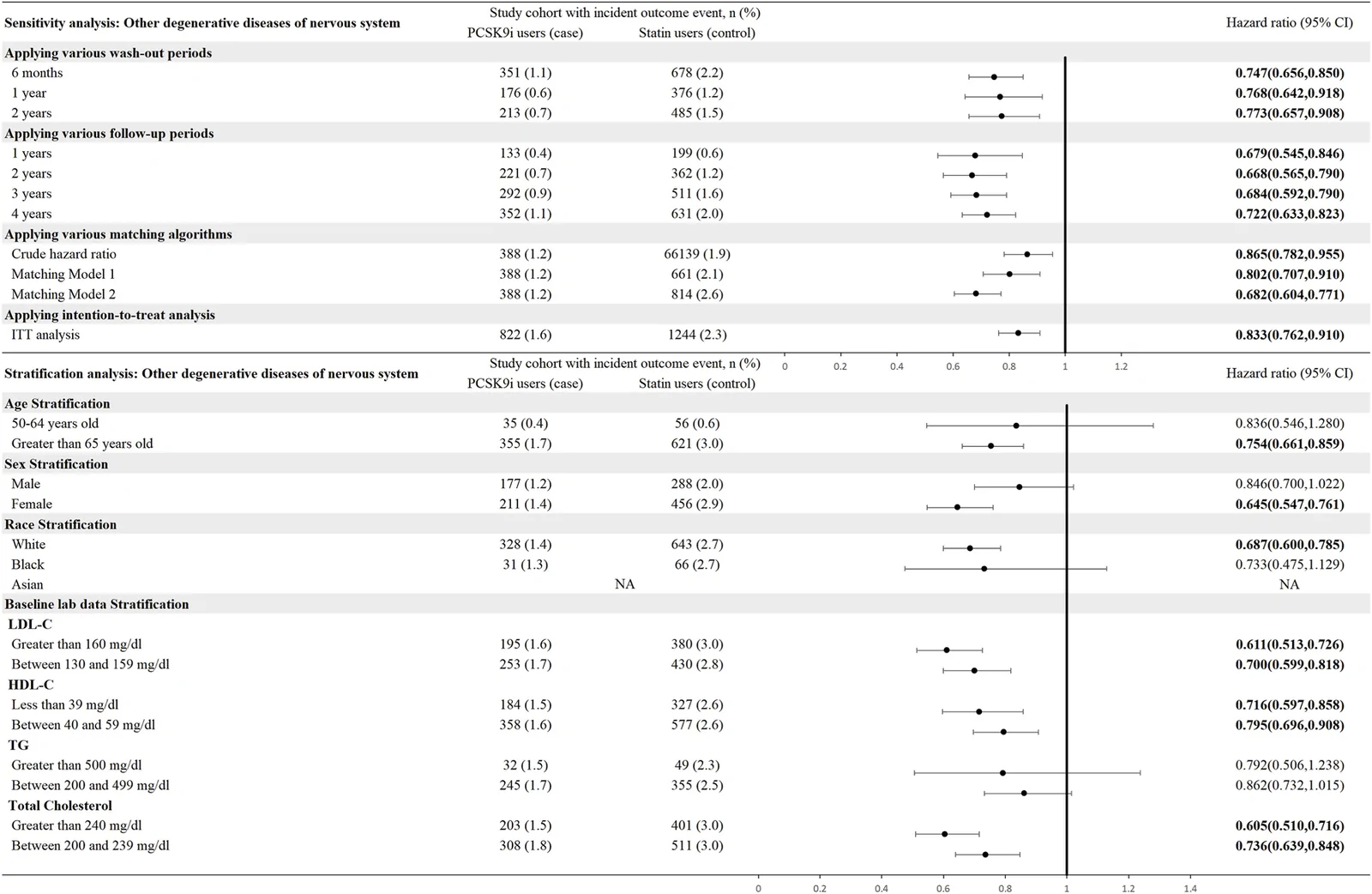
Risk of other degenerative diseases of the nervous system in different sensitivity and stratification models comparing the PCSK9 inhibitor Group and Statin Group.

In the primary matched cohorts, median follow-up was 782 days (IQR 945 days) for PCSK9 inhibitor users and 1,415 days (IQR 1,133 days) for statin users. Exact person-time and outcome-specific incidence rates were not available from the aggregate TriNetX output; event counts and cumulative cohort proportions are therefore reported.

In the statin-unrestricted sensitivity analysis, 54,387 patients were retained in each group after matching. All categorical lipid indicators and concomitant medications included in the expanded propensity-score model had post-matching standardized mean differences below 0.10, although some continuous descriptive laboratory measures remained imbalanced. PCSK9 inhibitor use remained associated with lower observed risks of all-cause dementia (HR 0.529; 95% CI 0.482–0.580), vascular dementia (HR 0.561; 95% CI 0.462–0.681), Alzheimer’s disease (HR 0.585; 95% CI 0.494–0.693), and other degenerative diseases of the nervous system (HR 0.806; 95% CI 0.742–0.876); the association with Parkinson’s disease was not statistically significant (HR 0.957; 95% CI 0.808–1.135) (Supplementary Tables S20–S21). Analyses retaining patients with recorded death produced directionally similar estimates in both the restricted-exposure and as-started frameworks (Supplementary Table S22).

### Subgroup and stratification analyses

Subgroup analyses demonstrated broadly consistent associations between PCSK9 inhibitors use and reduced dementia risk across major demographic strata (Supplementary Tables S23–S29; Figures 3–7). Risk reductions for all-cause dementia, vascular dementia, and Alzheimer’s disease were observed in both age groups (50–64 and ≥65 years), in women and men, and among White and Black participants. Effect estimates were generally more pronounced in women and older individuals, whereas estimates in younger and non-White subgroups were less precise, likely reflecting smaller numbers of events. Baseline lipid levels did not materially modify the associations (Supplementary Tables S30–S37; Figures 3–7). Across strata of LDL-C, HDL-C, and triglycerides, HRs for all-cause dementia typically ranged from 0.3 to 0.5. Notably, among participants with LDL-C ≥160 mg/dL, the association with Alzheimer’s disease was particularly strong (HR: 0.250; 95% CI: 0.167–0.376; Figure 5). In contrast, subgroup and lipid-stratified analyses for Parkinson’s disease were more heterogeneous, with wide confidence intervals frequently crossing the unity threshold.

## Discussion

In this large-scale emulated target trial, initiation of PCSK9 inhibitors was associated with a lower risk of incident dementia and major neurodegenerative disorders compared with statins and other lipid-lowering therapies, with relative risk reductions consistently ranging from approximately 45%–55% across all-cause dementia, vascular dementia, and Alzheimer’s disease. These associations remained robust in cross-dataset validation and a range of sensitivity analyses, and were consistently observed across different exposure definitions as well as demographic and clinical subgroups. The overall pattern of findings supports a potential association between PCSK9 inhibition and a lower risk of neurodegenerative outcomes.

The magnitude of risk reduction observed in this study exceeds that reported in randomized trials and meta-analyses evaluating neurocognitive outcomes with PCSK9 inhibition (Hirsh et al., 2021; Giugliano et al., 2017; Goldstein et al., 2023; Orringer et al., 2017; Reiffel, 2020; Rivera et al., 2024; Barbati et al., 2024; Bell et al., 2022; Small et al., 2020). The EBBINGHAUS substudy of FOURIER found no significant difference in cognitive outcomes between evolocumab and placebo over a median follow-up of 19 months, and meta-analyses have generally reported neutral effects on neurocognitive adverse events (Giugliano et al., 2017). Mendelian randomization findings are mixed. Earlier Mendelian randomization work found no evidence that lifelong lower LDL-C through HMGCR increased the risk of Alzheimer’s disease, vascular dementia, all-cause dementia, or Parkinson’s disease, with point estimates for HMGCR trending toward a protective effect on Parkinson’s disease (OR 0.83, 95% CI 0.65–1.07) though this crossed the null (Williams et al., 2020a). For PCSK9, the evidence is conflicting: one Mendelian randomization study found genetically lower LDL-C *via* PCSK9 was associated with a significantly increased risk of Alzheimer’s disease (OR 1.45, 95% CI 1.23–1.69) (Williams et al., 2020b), while a separate analysis of genetically proxied PCSK9 inhibition found a neutral effect on cognitive decline and dementia progression biomarkers, without evidence of harm (Rosoff et al., 2022). A subsequent analysis of more than one million individuals found a neutral estimate for genetically proxied PCSK9-mediated lowering of non-HDL-C and dementia, but protective associations for several other lipid-lowering targets (Nordestgaard et al., 2025). These genetic data neither establish a neuroprotective effect of pharmacologic PCSK9 inhibition nor suggest a clear increase in risk. The apparent discrepancy remains unresolved, and the available literature does not consistently support a reduction in Alzheimer’s disease risk associated with PCSK9 inhibition.

Several methodological considerations may explain these apparent discrepancies. Most randomized trials and genetic studies were not designed to assess incident dementia as a primary endpoint and were limited by relatively short follow-up durations and low event rates for neurodegenerative outcomes (Giugliano et al., 2017; Goldstein et al., 2023; Rosoff et al., 2022). Given that dementia typically develops over decades, follow-up periods of 1–3 years may be insufficient to detect meaningful differences in disease incidence (Giugliano et al., 2017; Goldstein et al., 2023; Williams et al., 2020b). The observed associations are biologically plausible in light of emerging evidence implicating PCSK9 in central nervous system cholesterol homeostasis, neuroinflammation, and amyloid-β (Aβ) metabolism (Bell et al., 2023b; Zheng et al., 2025; Zhu et al., 2024; O’Connell and Lohoff, 2020; Zhang et al., 2025; Mazura et al., 2022; Vilella et al., 2024; Papotti et al., 2022). PCSK9 is expressed in the brain and regulates the degradation of multiple LDL receptor family members, including LDLR and LRP1, which play key roles in Aβ clearance across the blood–brain barrier (Bell et al., 2023b; O’Connell and Lohoff, 2020; Mazura et al., 2022). Experimental studies demonstrate that PCSK9 inhibition enhances LRP1-mediated efflux of Aβ from the brain, resulting in reduced cerebral amyloid burden (Mazura et al., 2022). In transgenic Alzheimer’s disease mouse models, genetic ablation of PCSK9 attenuates Aβ deposition, suppresses neuroinflammatory signaling, and improves cognitive performance (Vilella et al., 2024). PCSK9 also modulates astrocyte-derived cholesterol trafficking to neurons, and excessive PCSK9 activity may reduce neuronal cholesterol availability, thereby exacerbating synaptic dysfunction and Aβ-related neurotoxicity (Papotti et al., 2022). In addition, PCSK9-mediated degradation of ApoER2 and related receptors may impair Reelin signaling, which is essential for synaptic plasticity and memory formation (Bell et al., 2023b; O’Connell and Lohoff, 2020). Preservation of these receptors through PCSK9 inhibition may influence amyloid metabolism, although these mechanisms remain to be confirmed in humans.

Other pathways may also contribute. PCSK9 inhibition may reduce systemic or vascular inflammation and improve endothelial nitric oxide bioavailability, which could in turn lessen cerebral microvascular injury (Imran et al., 2023; Pu et al., 2026). Improved endothelial function may help maintain blood-brain barrier integrity and reduce the entry of circulating inflammatory mediators (Sorond et al., 2026). Within the central nervous system, PCSK9 may influence LDL receptor-family proteins that participate in neuronal lipid uptake and Reelin signaling (Poirier et al., 2008). These proteins have also been implicated in amyloid-β processing (B et al., 2006). Changes in cholesterol trafficking between astrocytes and neurons could provide another link to synaptic function (Kim and Halliday, 2026). However, currently available PCSK9 monoclonal antibodies have limited penetration across an intact blood-brain barrier (Schonck et al., 2024). Any potential neurologic effects may therefore arise mainly from peripheral vascular changes, although context-dependent central signaling cannot be excluded. These mechanisms remain biologically plausible and should not be interpreted as established mediators of the associations observed in our study.

The pronounced reduction in vascular dementia observed in this study likely reflects both direct cerebrovascular protection and indirect modification of shared vascular risk factors. Hypertension, diabetes, dyslipidemia, and atherosclerosis are well-established causal contributors to both vascular cognitive impairment and Alzheimer’s disease (Iadecola et al., 2019; Iadecola et al., 2023; Smith et al., 2025). Beyond LDL-C lowering, PCSK9 inhibitors exert pleiotropic effects including improvement in endothelial function, reduction in vascular inflammation, and modulation of platelet activity (Goldstein et al., 2023; Orringer et al., 2017). These effects may reduce cerebral small-vessel disease burden and microvascular injury, which are increasingly recognized as key drivers of cognitive decline (Iadecola et al., 2019; Iadecola et al., 2023; Smith et al., 2025). The consistency of associations across vascular dementia and Alzheimer’s disease supports the concept of overlapping vascular and neurodegenerative pathways contributing to late-life cognitive impairment (Iadecola et al., 2019; Iadecola et al., 2023).

Associations between PCSK9 inhibitors use and Parkinson’s disease were less consistent than those observed for dementia. While a significant risk reduction was observed in the primary analysis, this association attenuated in several sensitivity and subgroup analyses, suggesting heterogeneity of effect. Parkinson’s disease pathogenesis is driven primarily by α-synuclein aggregation, mitochondrial dysfunction, and dopaminergic neuronal loss, processes that may be less sensitive to PCSK9-mediated lipid and vascular pathways (O’Connell and Lohoff, 2020; Zhang et al., 2025). In summary, these findings highlight the possibility that PCSK9 inhibition may preferentially influence neurodegenerative disorders with a substantial vascular or cholesterol-related component.

A major strength of this study is the target trial emulation framework, supported by active comparators, new-user eligibility, multiple sensitivity analyses, and external validation. Nevertheless, the findings remain observational and residual confounding is a major limitation. The electronic health records did not reliably capture APOE genotype, educational attainment, detailed smoking or alcohol exposure, physical activity, diet, baseline cognitive performance, family history of dementia, medication adherence, or the clinical severity prompting PCSK9 inhibitor initiation. Confounding by indication is particularly relevant because PCSK9 inhibitor recipients may differ in statin tolerance, insurance access, specialist involvement, follow-up intensity, adherence, and preventive care. The primary matching model balanced its prespecified covariates but did not include every descriptive laboratory or medication variable shown in Table 1; several of those variables remained imbalanced. The statin-unrestricted sensitivity analysis with an expanded matching model reduced imbalance in categorical lipid and medication covariates, but it cannot eliminate unmeasured confounding or imbalance in all continuous measurements. Exposure was based on recorded prescriptions. Minimum treatment duration, refill continuity, discontinuation, persistence, adherence, and intermittent use could not be fully ascertained. Excluding crossover in the primary restricted-exposure analysis may introduce post-index selection, whereas the as-started sensitivity analysis may dilute effects through subsequent switching. Formal proportional-hazards diagnostics and competing-risk regression were unavailable in the platform; analyses across shorter follow-up windows and analyses retaining recorded deaths partly assessed robustness but do not replace those methods. Follow-up was short relative to the long preclinical course of dementia, and diagnostic coding may miss cognitive decline or misclassify overlapping dementia subtypes. Finally, TriNetX predominantly represents healthcare systems in developed countries. Generalizability may therefore be limited for lower-income populations, underrepresented racial and ethnic groups, and settings with different access, reimbursement, or prescribing practices. The magnitude of the observed associations should consequently not be interpreted as a causal treatment effect.

These findings may inform future research on neurodegenerative outcomes among patients receiving intensive lipid-lowering therapy, but they do not support prescribing PCSK9 inhibitors for dementia prevention. PCSK9 inhibitors remain indicated for cardiovascular risk reduction in selected patients who do not achieve lipid targets with maximally tolerated therapy (Orringer et al., 2017). Prospective studies should include dementia-specific endpoints, longer follow-up, serial cognitive assessment, and biomarkers. Inclusion of patients with cognitive impairment, underrepresented populations, and genetic risk stratification may help clarify heterogeneity. Mechanistic studies integrating neuroimaging, cerebrospinal fluid biomarkers, and measures of blood-brain barrier integrity are also needed.

In conclusion, PCSK9 inhibitor use was associated with a lower observed risk of dementia and several neurodegenerative outcomes compared with conventional lipid-lowering therapies. The consistency across analyses supports the neurocognitive safety of intensive LDL-C lowering, but the observational design, residual confounding, exposure misclassification, and limited follow-up preclude causal or therapeutic claims. Long-term prospective studies with prespecified cognitive outcomes are needed to determine whether the association reflects a treatment effect.

## Data Availability

The data analyzed in this study is subject to the following licenses/restrictions: Data in this study were retrieved from TriNetX Research Network. All data available in the database were administrated by the TriNetX platform. Detailed information can be retrieved at the official website of the research network (https://trinetx.com). Requests to access these datasets should be directed to TriNetX research network (https://trinetx.com).
