# Supplementary material for: PCSK9 inhibitor use and risk of dementia and neurodegenerative diseases in adults aged 50 Years and older: a global electronic health record study
Source: Front Pharmacol. 2026 Oct 29;17:1942691. doi: 10.3389/fphar.2026.1942691 (PMC13755295; doi:10.3389/fphar.2026.1942691)
Supplement: Supplementary file 1 [file Table1.docx]

**Supplementary Files**

**Supplementary Tables**

**Table S1. Utilized administrative codes for disease and medication definition ^a^**

| **Description** | **Corresponding administrative codes** |
| --- | --- |
| *Study population* |  |
| Hyperlipidemia | ICD-10-CM E78 |
| *Interventions* |  |
| PCSK9 inhibitors | RxNorm codes 1659152 (alirocumab), 1665684 (evolocumab), 2588243 (inclisiran) |
| PCSK9 inhibitors monoclonal antibodies (mAbs) | RxNorm codes 1659152 (alirocumab), 1665684 (evolocumab) |
| PCSK9 inhibitors small interfering RNA (siRNA) | RxNorm codes 2588243 (inclisiran) |
| Statins | ATC code C10AA (HMG CoA reductase inhibitors) |
| Ezetimibe | RxNorm code 341248 (ezetimibe) |
| Fenofibrates | RxNorm code 4719 (gemfibrozil), 8703 (fenofibrate) |
| Cholestyramine | RxNorm code 2447 (cholestyramine resin) |
| *Main outcome event* |  |
| All-caused dementia | ICD-10-CM: F01-F03 |
| Vascular dementia | ICD-10-CM: F01 |
| Parkinson’s disease | ICD-10-CM: G20 |
| Alzheimer’s disease | ICD-10-CM: G30 |
| Other degenerative diseases of the nervous system | ICD-10-CM: G30-G32 |
| *Comorbidities and other definitions* |  |
| Essential hypertension | ICD-10-CM: I10 |
| Chronic ischemic heart disease | ICD-10-CM: I25 |
| Diabetes mellitus | ICD-10-CM: E08-E13 |
| Vitamin D deficiency | ICD-10-CM: E55 |
| Chronic kidney disease | ICD-10-CM: N18 |
| Diseases of liver | ICD-10-CM: K70-K77 |
| Cerebral infarction | ICD-10-CM: I63 |
| Persons with potential health hazards related to socioeconomic and psychosocial circumstances | ICD-10-CM: Z55-Z65 |
| Mental and behavioral disorders due to psychoactive substance use | ICD-10-CM: F10-F19 |
| *Comedications* |  |
| Vasoprotectives | ATC code: C05 |
| Beta blocking agents | ATC code: C07 |
| Agents acting on the renin-angiotensin system | ATC code: C09 |
| Aspirin | RxNorm code 1191 |
| Corticosteroids for systemic use | ATC code: H02 |
| Diuretics | ATC code: C03 |
| Calcium channel blockers | ATC code: C08 |
| Vitamins | VA code: VT000 |
| Immunosuppressants | ATC code: L04 |
| Niacins | RxNorm code 7393 |

^a^ICD-10-CM: International Classification of Diseases, Tenth Revision, Clinical Modification; ATC code, Anatomical Therapeutic Chemical Classification System; VA code, Veterans Affairs Drug Classification

**Table S2.** Comparative outlining of the idealized target trial framework and its implementation using observational data.

| **Component** | **Target trial** | **Current emulated trial using real-world data** |
| --- | --- | --- |
| **Primary aims of the investigation** | Study was designed to directly contrast the incidence of neurodegenerative conditions including all-cause dementia, vascular dementia, Alzheimer’s disease, Parkinson’s disease, and other degenerative disorders affecting the nervous system between individuals with hyperlipidemia treated with PCSK9 inhibitors and those receiving alternative lipid-lowering therapies such as statins, ezetimibe, fenofibrates, or cholestyramine. | Same |
| **Definition of inclusion standards for participant selection** | The study population comprised individuals aged 50 years and above with a documented history of hyperlipidemia, who had no prior diagnosis of cancer and no recorded death from any cause before cohort entry. | Same |
| **Classification of participants by therapeutic exposure** | Patients diagnosed with hyperlipidemia were categorized according to the lipid-lowering therapy they received. The exposure cohort consisted of individuals treated with PCSK9 inhibitors, while comparison cohorts included patients managed with alternative agents, namely statins, ezetimibe, fenofibrates, or cholestyramine. | Same |
| **Characterization of the analytical population and treatment assignment strategy** | Once eligibility was established, individuals were distributed among the intervention groups using a chance-based randomization procedure designed to balance assignment probability across all study arms. | Within the emulation framework, a propensity score–based matching strategy was applied to construct comparable exposure groups by equalizing the estimated likelihood of treatment assignment between the intervention and comparison cohorts. |
| **Definition of study initiation and termination points** | Follow-up began at the time of treatment allocation and continued until either the study outcome was first observed or the participant reached the predefined conclusion of the observation period, whichever occurred sooner. | Same |
| **Outcome event** | Incident neurodegenerative conditions including all-cause dementia, vascular dementia, Alzheimer’s disease, Parkinson’s disease, and other degenerative disorders affecting the nervous system | Same |
| **Models and effect** | The primary analytical approach followed a per-protocol framework, focusing on estimating the effect of continuous and consistent compliance with the prespecified treatment regimen across the full duration of follow-up. In contrast, sensitivity analyses were conducted using an intention-to-treat strategy, whereby all participants were analyzed according to their initial treatment assignment, capturing overall effectiveness irrespective of later treatment discontinuation, switching, or nonadherence. | The primary restricted-exposure analysis excluded individuals with documented crossover to the opposing therapy during follow-up. Because time-varying censoring with inverse-probability adjustment at the exact switching date was not available within the TriNetX interface, this analysis should not be interpreted as a fully adjusted per-protocol effect estimate. An as-started sensitivity analysis retained participants according to their initial exposure group regardless of subsequent treatment changes. |

*PCSK9 inhibitors, Proprotein Convertase Subtilisin/Kexin Type 9 Inhibitor*

**Table S3. Description of all applied sensitivity analysis models**

| **Models** | **Description** |
| --- | --- |
| **Applying different lagging periods after index date** | |
| 6 months/12 months /24 months after index date | In this series of sensitivity analyses, events arising during the prespecified lagging period were excluded from all subsequent statistical evaluations, while a standardized follow-up duration of five years was consistently applied to every subgroup examined. |
| 1 years /2 years /3 years / 4 years after index date | In this series of sensitivity analysis, event inclusion was restricted to outcomes verified within the predefined follow-up period, and a uniform one-month lagging period was applied across all analytical groups. |
| **Applying different matching covariates** | |
| Crude model | In this analysis, the initial hazard ratio derived from the full study population before any propensity score–based adjustment was evaluated |
| Matching model 1 | The matching process incorporated the covariates of participants’ age and sex. |
| Matching model 2 | The matching process incorporated not only the covariates specified in the primary analysis but also a comprehensive set of concomitant medications. These additional factors included vasoprotective agents, beta-adrenergic blockers, renin–angiotensin system inhibitors, aspirin, corticosteroids, diuretics, calcium channel blockers, vitamin supplements, immunosuppressive therapies, and niacin. |
| **Intention-to-treat (ITT) analysis** | |
| ITT analysis | This investigation followed an intention-to-treat analytic strategy within a target trial emulation design to compare the effects of PCSK9 inhibitors and active comparator treatments on the incidence of outcome events. Participants were analyzed according to their original treatment assignment (PCSK9 inhibitors, statins, ezetimibe, fenofibrates, or cholestyramine) without regard to treatment interruption, switching, or crossover to alternative lipid-lowering therapies. |

**Table S4**. Baseline characteristics of study population (before and after propensity score matching) in Emulated Trial 2 (PCSK9 inhibitors versus Ezetimibe)

|  | Before matching | | |  | After matching^a^ | | |
| --- | --- | --- | --- | --- | --- | --- | --- |
|  | PCSK9 inhibitors group  (n= 35,399) | Ezetimibe group  (n= 260,637) | SMD |  | PCSK9 inhibitors group  (n= 35,399) | Ezetimibe group  (n= 35,399) | SMD |
| **Age at index** |  |  |  |  |  |  |  |
| Mean±SD | 65.3±9.1 | 65.4±10.0 | 0.01 |  | 65.3±9.1 | 65.4±9.2 | 0.01 |
| **Sex** |  |  |  |  |  |  |  |
| Male | 18199 (51.4) | 139782 (53.6) | 0.04 |  | 18199 (51.4) | 18296 (51.7) | 0.01 |
| Female | 17142 (48.4) | 120591 (46.3) | 0.04 |  | 17142 (48.4) | 17056 (48.2) | 0.01 |
| Unknown Gender | 58 (<0.1) | 264 (<0.1) | 0.02 |  | 58 (<0.1) | 47 (<0.1) | 0.01 |
| **Race, n (%)** |  |  |  |  |  |  |  |
| White | 28037 (79.2) | 166140 (63.7) | 0.35 |  | 28037 (79.2) | 28385 (80.2) | 0.02 |
| Black or African American | 2996 (8.5) | 25352 (9.7) | 0.04 |  | 2996 (8.5) | 2967 (8.4) | <0.1 |
| Asian | 969 (2.7) | 11340 (4.4) | 0.09 |  | 969 (2.7) | 937 (2.7) | 0.01 |
| American Indian or Alaska Native | 158 (0.5) | 737 (0.3) | 0.03 |  | 158 (0.5) | 101 (0.3) | 0.03 |
| Native Hawaiian or other Pacific Islander | 90 (0.3) | 712 (0.3) | <0.1 |  | 90 (0.3) | 59 (0.2) | 0.02 |
| Other Race | 1116 (3.2) | 7089 (2.7) | 0.03 |  | 1116 (3.2) | 1007 (2.9) | 0.02 |
| Unknown Race | 2033 (5.7) | 49267 (18.9) | 0.41 |  | 2033 (5.7) | 1943 (5.5) | 0.01 |
| **Baseline lipid profile**, n (%) |  |  |  |  |  |  |  |
| Triglyceride in Serum, Plasma or Blood |  |  |  |  |  |  |  |
| ≧ 200 mg/dL | 9861 (27.9) | 55606 (21.3) | 0.15 |  | 9861 (27.9) | 8200 (23.2) | 0.11 |
| Cholesterol in HDL in Serum or Plasma |  |  |  |  |  |  |  |
| ≤ 40 mg/dL | 10141 (28.7) | 64420 (24.7) | 0.09 |  | 10141 (28.7) | 9891 (27.9) | 0.02 |
| Cholesterol in LDL in Serum or Plasma |  |  |  |  |  |  |  |
| ≧ 130 mg/dL | 14545 (41.1) | 79168 (30.4) | 0.22 |  | 14545 (41.1) | 11303 (31.9) | 0.19 |
| Cholesterol in Serum or Plasma |  |  |  |  |  |  |  |
| ≧ 200 mg/dL | 15948 (45.1) | 89389 (34.3) | 0.22 |  | 15948 (45.1) | 12913 (36.5) | 0.18 |
| **Other lab data profile**, n (%) |  |  |  |  |  |  |  |
| Urate level in Serum or Plasma |  |  |  |  |  |  |  |
| ≧ 7.5 mg/dL | 946 (2.7) | 9777 (3.8) | 0.06 |  | 946 (2.7) | 915 (2.6) | 0.01 |
| HbA1c |  |  |  |  |  |  |  |
| ≧ 5.7% | 12809 (36.2) | 94516 (36.3) | <0.1 |  | 12809 (36.2) | 12834 (36.3) | <0.1 |
| **BMI,** n (%) |  |  |  |  |  |  |  |
| ≧ 25 (kg/m^2^) | 21255 (60.0) | 124977 (48.0) | 0.24 |  | 21255 (60.0) | 21238 (60.0) | <0.1 |
| **Medical Utilization status** |  |  |  |  |  |  |  |
| Visit: Ambulatory | 29380 (83.0) | 202679 (77.8) | 0.13 |  | 29380 (83.0) | 29416 (83.1) | <0.1 |
| Visit: Inpatient Encounter | 10390 (29.4) | 78740 (30.2) | 0.02 |  | 10390 (29.4) | 10460 (29.6) | <0.1 |
| **Socioeconomic status** |  |  |  |  |  |  |  |
| Persons with potential health hazards related to socioeconomic and psychosocial circumstances | 612 (1.7) | 4239 (1.6) | 0.01 |  | 612 (1.7) | 431 (1.2) | 0.04 |
| **Family history** |  |  |  |  |  |  |  |
| Family history of other mental and behavioral disorders | 79 (0.2) | 435 (0.2) | 0.01 |  | 79 (0.2) | 49 (0.1) | 0.02 |
| Family history of other endocrine, nutritional and metabolic diseases | 242 (0.7) | 1102 (0.4) | 0.04 |  | 242 (0.7) | 213 (0.6) | 0.01 |
| **Lifestyle** |  |  |  |  |  |  |  |
| Mental and behavioral disorders due to psychoactive substance use | 5188 (14.7) | 33374 (12.8) | 0.05 |  | 5188 (14.7) | 4938 (14.0) | 0.02 |
| **Comorbidities** |  |  |  |  |  |  |  |
| Essential hypertension | 24311 (68.7) | 160993 (61.8) | 0.15 |  | 24311 (68.7) | 24311 (68.7) | <0.1 |
| Chronic ischemic heart disease | 19700 (55.7) | 102053 (39.2) | 0.33 |  | 19700 (55.7) | 19581 (55.3) | 0.01 |
| Diabetes mellitus | 10718 (30.3) | 86051 (33.0) | 0.06 |  | 10718 (30.3) | 10580 (29.9) | 0.01 |
| Vitamin D deficiency | 6012 (17.0) | 36621 (14.1) | 0.08 |  | 6012 (17.0) | 5718 (16.2) | 0.02 |
| Chronic kidney disease | 3972 (11.2) | 30714 (11.8) | 0.02 |  | 3972 (11.2) | 3665 (10.4) | 0.03 |
| Diseases of liver | 3028 (8.6) | 17915 (6.9) | 0.06 |  | 3028 (8.6) | 2383 (6.7) | 0.07 |
| Cerebral infarction | 2252 (6.4) | 15374 (5.9) | 0.02 |  | 2252 (6.4) | 1975 (5.6) | 0.03 |
| **Comedications** |  |  |  |  |  |  |  |
| Vasoprotectives | 21615 (61.1) | 126677 (48.6) | 0.25 |  | 21615 (61.1) | 19550 (55.2) | 0.12 |
| Beta blockers | 19080 (53.9) | 108729 (41.7) | 0.25 |  | 19080 (53.9) | 17191 (48.6) | 0.11 |
| Agents acting on the renin-angiotensin system | 18154 (51.3) | 112753 (43.3) | 0.16 |  | 18154 (51.3) | 16593 (46.9) | 0.09 |
| Aspirin | 17735 (50.1) | 97890 (37.6) | 0.25 |  | 17735 (50.1) | 15520 (43.8) | 0.13 |
| Corticosteroids | 16223 (45.8) | 92901 (35.6) | 0.21 |  | 16223 (45.8) | 14253 (40.3) | 0.11 |
| Diuretics | 13481 (38.1) | 78047 (30.0) | 0.17 |  | 13481 (38.1) | 11952 (33.8) | 0.09 |
| Calcium channel blockers | 13078 (37.0) | 77491 (29.7) | 0.15 |  | 13078 (37.0) | 11101 (31.4) | 0.12 |
| Vitamins | 11394 (32.2) | 59661 (22.9) | 0.21 |  | 11394 (32.2) | 9061 (25.6) | 0.15 |
| Immunosuppressants | 1668 (4.7) | 7338 (2.8) | 0.1 |  | 1668 (4.7) | 1098 (3.1) | 0.08 |
| Niacin | 1166 (3.3) | 6061 (2.3) | 0.06 |  | 1166 (3.3) | 852 (2.4) | 0.05 |

PCSK9 inhibitors, Proprotein Convertase Subtilisin/Kexin Type 9 Inhibitor; HDL, High-Density Lipoprotein; LDL, Low-Density Lipoprotein; BMI, body mass index; SD, standardized difference; SMD, standardized mean difference. A standardized mean difference (SMD) exceeding 0.1 was used to define a clinically relevant difference between comparison groups. In the TriNetX analytics platform, to maintain patient deidentification, incident case counts of 10 or fewer are censored and displayed as 10. Propensity score matching was implemented while controlling for baseline demographic variables (index age, sex, and race), body mass index, socioeconomic and lifestyle characteristics (socioeconomic or psychosocial–related health hazards, Family history of other mental and behavioral disorders (Z81.8), and mental or behavioral disorders associated with psychoactive substance use), healthcare utilization patterns (ambulatory and inpatient encounters), and a set of prespecified comorbidities, including essential hypertension, diabetes mellitus chronic ischemic heart disease, cerebral infarction, chronic kidney disease, and vitamin D deficiency.

**Table S5**. Baseline characteristics of study population (before and after propensity score matching) in Emulated Trial 3 (PCSK9 inhibitors versus Fenofibrates)

|  | Before matching | | |  | After matching^a^ | | |
| --- | --- | --- | --- | --- | --- | --- | --- |
|  | PCSK9 inhibitors group  (n= 50,134) | Fenofibrates group  (n= 195,432) | SMD |  | PCSK9 inhibitors group  (n= 43,629) | Fenofibrates group  (n= 43,629) | SMD |
| **Age at index** |  |  |  |  |  |  |  |
| Mean±SD | 65.1±9.2 | 60.6±10.5 | 0.46 |  | 64.5±9.1 | 65.1±9.9 | 0.07 |
| **Sex** |  |  |  |  |  |  |  |
| Male | 25481 (50.8) | 123081 (63.0) | 0.25 |  | 23227 (53.2) | 22780 (52.2) | 0.02 |
| Female | 24580 (49.0) | 72200 (36.9) | 0.25 |  | 20340 (46.6) | 20782 (47.6) | 0.02 |
| Unknown Gender | 73 (0.2) | 151 (<0.1) | 0.02 |  | 62 (0.1) | 67 (0.2) | 0.00 |
| **Race, n (%)** |  |  |  |  |  |  |  |
| White | 39235 (78.3) | 133149 (68.1) | 0.23 |  | 33914 (77.7) | 34384 (78.8) | 0.03 |
| Black or African American | 4647 (9.3) | 9944 (5.1) | 0.16 |  | 3715 (8.5) | 3574 (8.2) | 0.01 |
| Asian | 1385 (2.8) | 10197 (5.2) | 0.13 |  | 1354 (3.1) | 1309 (3.0) | 0.01 |
| American Indian or Alaska Native | 218 (0.4) | 736 (0.4) | 0.01 |  | 189 (0.4) | 178 (0.4) | 0.00 |
| Native Hawaiian or other Pacific Islander | 126 (0.3) | 675 (0.4) | 0.02 |  | 117 (0.3) | 112 (0.3) | 0.00 |
| Other Race | 1494 (3.0) | 5967 (3.1) | 0.00 |  | 1349 (3.1) | 1276 (2.9) | 0.01 |
| Unknown Race | 3029 (6.0) | 34764 (17.8) | 0.37 |  | 2991 (6.9) | 2796 (6.4) | 0.02 |
| **Baseline lipid profile**, n (%) |  |  |  |  |  |  |  |
| Triglyceride in Serum, Plasma or Blood |  |  |  |  |  |  |  |
| ≧ 200 mg/dL | 13832 (27.6) | 69110 (35.4) | 0.17 |  | 12223 (28.0) | 16441 (37.7) | 0.21 |
| Cholesterol in HDL in Serum or Plasma |  |  |  |  |  |  |  |
| ≤ 40 mg/dL | 14614 (29.2) | 62016 (31.7) | 0.06 |  | 12814 (29.4) | 15306 (35.1) | 0.12 |
| Cholesterol in LDL in Serum or Plasma |  |  |  |  |  |  |  |
| ≧ 130 mg/dL | 21950 (43.8) | 29427 (15.1) | 0.66 |  | 18921 (43.4) | 7691 (17.6) | 0.58 |
| Cholesterol in Serum or Plasma |  |  |  |  |  |  |  |
| ≧ 200 mg/dL | 23744 (47.4) | 51806 (26.5) | 0.44 |  | 20454 (46.9) | 12249 (28.1) | 0.40 |
| **Other lab data profile**, n (%) |  |  |  |  |  |  |  |
| Urate level in Serum or Plasma |  |  |  |  |  |  |  |
| ≧ 7.5 mg/dL | 1392 (2.8) | 6798 (3.5) | 0.04 |  | 1237 (2.8) | 1408 (3.2) | 0.02 |
| HbA1c |  |  |  |  |  |  |  |
| ≧ 5.7% | 18532 (37.0) | 63859 (32.7) | 0.09 |  | 16389 (37.6) | 15520 (35.6) | 0.04 |
| **BMI,** n (%) |  |  |  |  |  |  |  |
| ≧ 25 (kg/m^2^) | 29909 (59.7) | 85164 (43.6) | 0.33 |  | 25344 (58.1) | 25677 (58.9) | 0.02 |
| **Medical Utilization status** |  |  |  |  |  |  |  |
| Visit: Ambulatory | 41702 (83.2) | 141833 (72.6) | 0.26 |  | 35705 (81.8) | 35817 (82.1) | 0.01 |
| Visit: Inpatient Encounter | 15641 (31.2) | 43960 (22.5) | 0.20 |  | 13394 (30.7) | 13619 (31.2) | 0.01 |
| **Socioeconomic status** |  |  |  |  |  |  |  |
| Persons with potential health hazards related to socioeconomic and psychosocial circumstances | 903 (1.8) | 2514 (1.3) | 0.04 |  | 764 (1.8) | 709 (1.6) | 0.01 |
| **Family history** |  |  |  |  |  |  |  |
| Family history of other mental and behavioral disorders | 115 (0.2) | 329 (0.2) | 0.01 |  | 101 (0.2) | 89 (0.2) | 0.01 |
| Family history of other endocrine, nutritional and metabolic diseases | 347 (0.7) | 547 (0.3) | 0.06 |  | 300 (0.7) | 155 (0.4) | 0.05 |
| **Lifestyle** |  |  |  |  |  |  |  |
| Mental and behavioral disorders due to psychoactive substance use | 7577 (15.1) | 25294 (12.9) | 0.06 |  | 6708 (15.4) | 6798 (15.6) | 0.01 |
| **Comorbidities** |  |  |  |  |  |  |  |
| Essential hypertension | 34596 (69.0) | 104255 (53.4) | 0.33 |  | 29246 (67.0) | 29640 (67.9) | 0.02 |
| Chronic ischemic heart disease | 29237 (58.3) | 32710 (16.7) | 0.95 |  | 22738 (52.1) | 22677 (52.0) | 0.00 |
| Diabetes mellitus | 14952 (29.8) | 72209 (37.0) | 0.15 |  | 14062 (32.2) | 15259 (35.0) | 0.06 |
| Vitamin D deficiency | 8634 (17.2) | 22430 (11.5) | 0.16 |  | 7051 (16.2) | 7117 (16.3) | 0.00 |
| Chronic kidney disease | 5658 (11.3) | 18111 (9.3) | 0.07 |  | 5039 (11.6) | 5310 (12.2) | 0.02 |
| Diseases of liver | 4169 (8.3) | 14244 (7.3) | 0.04 |  | 3626 (8.3) | 3152 (7.2) | 0.04 |
| Cerebral infarction | 3311 (6.6) | 6635 (3.4) | 0.15 |  | 2651 (6.1) | 2768 (6.3) | 0.01 |
| **Comedications** |  |  |  |  |  |  |  |
| Vasoprotectives | 31277 (62.4) | 67153 (34.4) | 0.58 |  | 26491 (60.7) | 21290 (48.8) | 0.24 |
| Beta blockers | 27882 (55.6) | 53482 (27.4) | 0.60 |  | 23404 (53.6) | 18822 (43.1) | 0.21 |
| Agents acting on the renin-angiotensin system | 26240 (52.3) | 64413 (33.0) | 0.40 |  | 22543 (51.7) | 17891 (41.0) | 0.22 |
| Aspirin | 26331 (52.5) | 39395 (20.2) | 0.71 |  | 22157 (50.8) | 15108 (34.6) | 0.33 |
| Corticosteroids | 23010 (45.9) | 54786 (28.0) | 0.38 |  | 19623 (45.0) | 15506 (35.5) | 0.19 |
| Diuretics | 19439 (38.8) | 43300 (22.2) | 0.37 |  | 16538 (37.9) | 13386 (30.7) | 0.15 |
| Calcium channel blockers | 19045 (38.0) | 36472 (18.7) | 0.44 |  | 15995 (36.7) | 11288 (25.9) | 0.23 |
| Vitamins | 16387 (32.7) | 32819 (16.8) | 0.37 |  | 13948 (32.0) | 10018 (23.0) | 0.20 |
| Immunosuppressants | 2304 (4.6) | 3610 (1.9) | 0.16 |  | 2008 (4.6) | 991 (2.3) | 0.13 |
| Niacin | 1740 (3.5) | 4702 (2.4) | 0.06 |  | 1523 (3.5) | 1285 (3.0) | 0.03 |

PCSK9 inhibitors, Proprotein Convertase Subtilisin/Kexin Type 9 Inhibitor; HDL, High-Density Lipoprotein; LDL, Low-Density Lipoprotein; BMI, body mass index; SD, standardized difference; SMD, standardized mean difference. A standardized mean difference (SMD) exceeding 0.1 was used to define a clinically relevant difference between comparison groups. In the TriNetX analytics platform, to maintain patient deidentification, incident case counts of 10 or fewer are censored and displayed as 10. Propensity score matching was implemented while controlling for baseline demographic variables (index age, sex, and race), body mass index, socioeconomic and lifestyle characteristics (socioeconomic or psychosocial–related health hazards, Family history of other mental and behavioral disorders (Z81.8), and mental or behavioral disorders associated with psychoactive substance use), healthcare utilization patterns (ambulatory and inpatient encounters), and a set of prespecified comorbidities, including essential hypertension, diabetes mellitus chronic ischemic heart disease, cerebral infarction, chronic kidney disease, and vitamin D deficiency.

**Table S6**. Baseline characteristics of study population (before and after propensity score matching) in Emulated Trial 4 (PCSK9 inhibitors versus Cholestyramine)

|  | Before matching | | |  | After matching^a^ | | |
| --- | --- | --- | --- | --- | --- | --- | --- |
|  | PCSK9 inhibitors group  (n= 53,027) | Cholestyramine group  (n= 27,433) | SMD |  | PCSK9 inhibitors group  (n= 23,163) | Cholestyramine group  (n= 23,163) | SMD |
| **Age at index** |  |  |  |  |  |  |  |
| Mean±SD | 64.9±9.2 | 66.1±11.4 | 0.12 |  | 65.8±9.5 | 65.6±11.4 | 0.01 |
| **Sex** |  |  |  |  |  |  |  |
| Male | 27328 (51.5) | 8790 (32.0) | 0.40 |  | 7958 (34.4) | 8142 (35.2) | 0.02 |
| Female | 25616 (48.3) | 18613 (67.8) | 0.40 |  | 15169 (65.5) | 14992 (64.7) | 0.02 |
| Unknown Gender | 83 (0.2) | 30 (0.1) | 0.01 |  | 36 (0.2) | 29 (0.1) | 0.01 |
| **Race, n (%)** |  |  |  |  |  |  |  |
| White | 41653 (78.6) | 22687 (82.7) | 0.11 |  | 18962 (81.9) | 18924 (81.7) | 0.00 |
| Black or African American | 4780 (9.0) | 2516 (9.2) | 0.01 |  | 2239 (9.7) | 2168 (9.4) | 0.01 |
| Asian | 1474 (2.8) | 430 (1.6) | 0.08 |  | 401 (1.7) | 408 (1.8) | 0.00 |
| American Indian or Alaska Native | 227 (0.4) | 112 (0.4) | 0.00 |  | 74 (0.3) | 88 (0.4) | 0.01 |
| Native Hawaiian or other Pacific Islander | 131 (0.2) | 49 (0.2) | 0.01 |  | 41 (0.2) | 44 (0.2) | 0.00 |
| Other Race | 1574 (3.0) | 620 (2.3) | 0.04 |  | 523 (2.3) | 572 (2.5) | 0.01 |
| Unknown Race | 3188 (6.0) | 1019 (3.7) | 0.11 |  | 923 (4.0) | 959 (4.1) | 0.01 |
| **Baseline lipid profile**, n (%) |  |  |  |  |  |  |  |
| Triglyceride in Serum, Plasma or Blood |  |  |  |  |  |  |  |
| ≧ 200 mg/dL | 15424 (29.1) | 6191 (22.6) | 0.15 |  | 7050 (30.4) | 5068 (21.9) | 0.20 |
| Cholesterol in HDL in Serum or Plasma |  |  |  |  |  |  |  |
| ≤ 40 mg/dL | 16028 (30.2) | 6800 (24.8) | 0.12 |  | 6142 (26.5) | 5644 (24.4) | 0.05 |
| Cholesterol in LDL in Serum or Plasma |  |  |  |  |  |  |  |
| ≧ 130 mg/dL | 23018 (43.4) | 6239 (22.7) | 0.45 |  | 10984 (47.4) | 5157 (22.3) | 0.55 |
| Cholesterol in Serum or Plasma |  |  |  |  |  |  |  |
| ≧ 200 mg/dL | 25195 (47.5) | 8157 (29.7) | 0.37 |  | 11879 (51.3) | 6689 (28.9) | 0.47 |
| **Other lab data profile**, n (%) |  |  |  |  |  |  |  |
| Urate level in Serum or Plasma |  |  |  |  |  |  |  |
| ≧ 7.5 mg/dL | 1507 (2.8) | 912 (3.3) | 0.03 |  | 684 (3.0) | 720 (3.1) | 0.01 |
| HbA1c |  |  |  |  |  |  |  |
| ≧ 5.7% | 19972 (37.7) | 9833 (35.8) | 0.04 |  | 9101 (39.3) | 7902 (34.1) | 0.11 |
| **BMI,** n (%) |  |  |  |  |  |  |  |
| ≧ 25 (kg/m^2^) | 31691 (59.8) | 16632 (60.6) | 0.02 |  | 13550 (58.5) | 13600 (58.7) | 0.00 |
| **Medical Utilization status** |  |  |  |  |  |  |  |
| Visit: Ambulatory | 44053 (83.1) | 23136 (84.3) | 0.03 |  | 19118 (82.5) | 19139 (82.6) | 0.00 |
| Visit: Inpatient Encounter | 16543 (31.2) | 11243 (41.0) | 0.20 |  | 7553 (32.6) | 7848 (33.9) | 0.03 |
| **Socioeconomic status** |  |  |  |  |  |  |  |
| Persons with potential health hazards related to socioeconomic and psychosocial circumstances | 958 (1.8) | 969 (3.5) | 0.11 |  | 581 (2.5) | 630 (2.7) | 0.01 |
| **Family history** |  |  |  |  |  |  |  |
| Family history of other mental and behavioral disorders | 131 (0.2) | 131 (0.5) | 0.04 |  | 80 (0.3) | 77 (0.3) | 0.00 |
| Family history of other endocrine, nutritional and metabolic diseases | 363 (0.7) | 248 (0.9) | 0.02 |  | 179 (0.8) | 197 (0.9) | 0.01 |
| **Lifestyle** |  |  |  |  |  |  |  |
| Mental and behavioral disorders due to psychoactive substance use | 8137 (15.3) | 4985 (18.2) | 0.08 |  | 3679 (15.9) | 3757 (16.2) | 0.01 |
| **Comorbidities** |  |  |  |  |  |  |  |
| Essential hypertension | 36660 (69.1) | 18046 (65.8) | 0.07 |  | 14923 (64.4) | 14773 (63.8) | 0.01 |
| Chronic ischemic heart disease | 16341 (30.8) | 9542 (34.8) | 0.08 |  | 7851 (33.9) | 7524 (32.5) | 0.03 |
| Diabetes mellitus | 30811 (58.1) | 6208 (22.6) | 0.78 |  | 6167 (26.6) | 6189 (26.7) | 0.00 |
| Vitamin D deficiency | 9175 (17.3) | 5980 (21.8) | 0.11 |  | 4959 (21.4) | 4792 (20.7) | 0.02 |
| Chronic kidney disease | 6099 (11.5) | 4760 (17.4) | 0.17 |  | 3425 (14.8) | 3409 (14.7) | 0.00 |
| Diseases of liver | 4503 (8.5) | 4060 (14.8) | 0.20 |  | 2152 (9.3) | 3226 (13.9) | 0.15 |
| Cerebral infarction | 3470 (6.5) | 1611 (5.9) | 0.03 |  | 1466 (6.3) | 1390 (6.0) | 0.01 |
| **Comedications** |  |  |  |  |  |  |  |
| Vasoprotectives | 33044 (62.3) | 17961 (65.5) | 0.07 |  | 13579 (58.6) | 14488 (62.5) | 0.08 |
| Beta blockers | 29629 (55.9) | 13188 (48.1) | 0.16 |  | 10960 (47.3) | 10724 (46.3) | 0.02 |
| Agents acting on the renin-angiotensin system | 27948 (52.7) | 12561 (45.8) | 0.14 |  | 11496 (49.6) | 10382 (44.8) | 0.10 |
| Aspirin | 27833 (52.5) | 9711 (35.4) | 0.35 |  | 10122 (43.7) | 8018 (34.6) | 0.19 |
| Corticosteroids | 24310 (45.8) | 16276 (59.3) | 0.27 |  | 11347 (49.0) | 13197 (57.0) | 0.16 |
| Diuretics | 20641 (38.9) | 11704 (42.7) | 0.08 |  | 9061 (39.1) | 9484 (40.9) | 0.04 |
| Calcium channel blockers | 20156 (38.0) | 8634 (31.5) | 0.14 |  | 8029 (34.7) | 7053 (30.4) | 0.09 |
| Vitamins | 17395 (32.8) | 10895 (39.7) | 0.14 |  | 8144 (35.2) | 8740 (37.7) | 0.05 |
| Immunosuppressants | 2435 (4.6) | 2051 (7.5) | 0.12 |  | 1235 (5.3) | 1634 (7.1) | 0.07 |
| Niacin | 1906 (3.6) | 525 (1.9) | 0.10 |  | 746 (3.2) | 444 (1.9) | 0.08 |

PCSK9 inhibitors, Proprotein Convertase Subtilisin/Kexin Type 9 Inhibitor; HDL, High-Density Lipoprotein; LDL, Low-Density Lipoprotein; BMI, body mass index; SD, standardized difference; SMD, standardized mean difference. A standardized mean difference (SMD) exceeding 0.1 was used to define a clinically relevant difference between comparison groups. In the TriNetX analytics platform, to maintain patient deidentification, incident case counts of 10 or fewer are censored and displayed as 10. Propensity score matching was implemented while controlling for baseline demographic variables (index age, sex, and race), body mass index, socioeconomic and lifestyle characteristics (socioeconomic or psychosocial–related health hazards, Family history of other mental and behavioral disorders (Z81.8), and mental or behavioral disorders associated with psychoactive substance use), healthcare utilization patterns (ambulatory and inpatient encounters), and a set of prespecified comorbidities, including essential hypertension, diabetes mellitus chronic ischemic heart disease, cerebral infarction, chronic kidney disease, and vitamin D deficiency.

**Table S7. Comparing PCSK9 inhibitors monoclonal antibodies (mAbs) with PCSK9 inhibitors small interfering RNA (siRNA)**

|  | Comparing mAbs versus siRNA | | |
| --- | --- | --- | --- |
| **Outcomes** | PCSK9 inhibitors monoclonal antibodies (mAbs) cohort (%)^a^ | PCSK9 inhibitors small interfering RNA (siRNA) cohort (%) | HR (95% CI)^a^ |
| All-caused dementia | 29 (1.7) | 11 (0.6) | 0.92 (0.415,2.037) |
| Vascular dementia | NA | NA | NA |
| Parkinson’s disease | NA | NA | NA |
| Alzheimer’s disease | NA | NA | NA |
| Other degenerative diseases of nervous system | 38 (2.2) | 21 (1.2) | 0.917 (0.518,1.626) |

PCSK9 inhibitors, Proprotein Convertase Subtilisin/Kexin Type 9 Inhibitor; In the TriNetX analytics platform, to maintain patient deidentification, incident case counts of 10 or fewer are censored and displayed as 10. Propensity score matching was implemented while controlling for baseline demographic variables (index age, sex, and race), body mass index, socioeconomic and lifestyle characteristics (socioeconomic or psychosocial–related health hazards, Family history of other mental and behavioral disorders (Z81.8), and mental or behavioral disorders associated with psychoactive substance use), healthcare utilization patterns (ambulatory and inpatient encounters), and a set of prespecified comorbidities, including essential hypertension, diabetes mellitus chronic ischemic heart disease, cerebral infarction, chronic kidney disease, and vitamin D deficiency.

**Table S8. Cross-dataset validation from the US collaborative network**

|  | Comparing with Statins users | | | Comparing with Ezetimibe users | | |
| --- | --- | --- | --- | --- | --- | --- |
| **Outcomes** | PCSK9 inhibitors cohort (%)^a^ | Ezetimibe cohort (%) | HR (95% CI)^a^ | PCSK9 inhibitors cohort (%)^a^ | Fenofibrates cohort (%) | HR (95% CI)^a^ |
| All-caused dementia | 211 (1.0) | 585 (2.8) | 0.424 (0.362,0.496) | 306 (1.1) | 474 (1.8) | 0.705 (0.61,0.814) |
| Vascular dementia | 50 (0.2) | 122 (0.6) | 0.49 (0.352,0.682) | 96 (0.4) | 146 (0.5) | 0.721 (0.557,0.933) |
| Parkinson’s disease | 72 (0.3) | 118 (0.6) | 0.721 (0.537,0.968) | 119 (0.4) | 131 (0.5) | 0.99 (0.772,1.27) |
| Alzheimer’s disease | 66 (0.3) | 179 (0.8) | 0.439 (0.331,0.583) | 69 (0.3) | 107 (0.4) | 0.714 (0.527,0.967) |
| Other degenerative diseases of nervous system | 316 (1.5) | 492 (2.3) | 0.763 (0.662,0.879) | 416 (1.5) | 512 (1.9) | 0.887 (0.78,1.01) |
|  | Comparing with Fenofibrates users | | | Comparing with Cholestyramine users | | |
| **Outcomes** | PCSK9 inhibitors cohort (%)^a^ | Ezetimibe cohort (%) | HR (95% CI)^a^ | PCSK9 inhibitors cohort (%)^a^ | Fenofibrates cohort (%) | HR (95% CI)^a^ |
| All-caused dementia | 386 (1.1) | 899 (2.6) | 0.487 (0.432,0.549) | 228 (1.3) | 592 (3.5) | 0.42 (0.36,0.489) |
| Vascular dementia | 92 (0.3) | 192 (0.6) | 0.545 (0.425,0.7) | 54 (0.3) | 101 (0.6) | 0.6 (0.431,0.836) |
| Parkinson’s disease | 148 (0.4) | 220 (0.6) | 0.761 (0.617,0.938) | 71 (0.4) | 152 (0.9) | 0.511 (0.385,0.678) |
| Alzheimer’s disease | 117 (0.3) | 264 (0.8) | 0.517 (0.415,0.643) | 62 (0.4) | 160 (0.9) | 0.425 (0.317,0.57) |
| Other degenerative diseases of nervous system | 576 (1.7) | 820 (2.4) | 0.812 (0.73,0.904) | 307 (1.8) | 565 (3.3) | 0.591 (0.514,0.679) |

In this analysis, the settings are the same as in the main analysis, adopting up to 5 years of follow-up and 1 month of wash-out period.

PCSK9 inhibitors, Proprotein Convertase Subtilisin/Kexin Type 9 Inhibitor; In the TriNetX analytics platform, to maintain patient deidentification, incident case counts of 10 or fewer are censored and displayed as 10. Propensity score matching was implemented while controlling for baseline demographic variables (index age, sex, and race), body mass index, socioeconomic and lifestyle characteristics (socioeconomic or psychosocial–related health hazards, Family history of other mental and behavioral disorders (Z81.8), and mental or behavioral disorders associated with psychoactive substance use), healthcare utilization patterns (ambulatory and inpatient encounters), and a set of prespecified comorbidities, including essential hypertension, diabetes mellitus chronic ischemic heart disease, cerebral infarction, chronic kidney disease, and vitamin D deficiency.

**Table S9. Sensitivity analysis: applying 6-months of lagging period after index date**

|  | Comparing with Ezetimibe users | | | Comparing with Fenofibrates users | | | Comparing with Cholestyramine users | | |
| --- | --- | --- | --- | --- | --- | --- | --- | --- | --- |
| **Outcomes** | PCSK9 inhibitors cohort (%)^a^ | Ezetimibe cohort (%) | HR (95% CI)^a^ | PCSK9 inhibitors cohort (%)^a^ | Fenofibrates cohort (%) | HR (95% CI)^a^ | PCSK9 inhibitors cohort (%)^a^ | Cholestyramine cohort (%) | HR (95% CI)^a^ |
| All-caused dementia | 337 (1.0) | 603 (1.7) | 0.652 (0.57,0.745) | 381 (0.9) | 1179 (2.7) | 0.425 (0.378,0.477) | 144 (0.6) | 422 (1.8) | 0.519 (0.43,0.627) |
| Vascular dementia | 79 (0.2) | 142 (0.4) | 0.657 (0.499,0.866) | 98 (0.2) | 245 (0.6) | 0.527 (0.416,0.667) | 29 (0.1) | 78 (0.3) | 0.572 (0.373,0.876) |
| Parkinson’s disease | 142 (0.4) | 176 (0.5) | 0.935 (0.749,1.167) | 183 (0.4) | 257 (0.6) | 0.934 (0.772,1.13) | 52 (0.2) | 130 (0.6) | 0.606 (0.439,0.837) |
| Alzheimer’s disease | 111 (0.3) | 178 (0.5) | 0.744 (0.587,0.943) | 113 (0.3) | 306 (0.7) | 0.491 (0.395,0.61) | 37 (0.2) | 110 (0.5) | 0.518 (0.357,0.752) |
| Other degenerative diseases of nervous system | 479 (1.4) | 617 (1.7) | 0.902 (0.8,1.017) | 550 (1.3) | 935 (2.2) | 0.772 (0.694,0.858) | 181 (0.8) | 369 (1.6) | 0.751 (0.628,0.897) |

PCSK9 inhibitors, Proprotein Convertase Subtilisin/Kexin Type 9 Inhibitor; In the TriNetX analytics platform, to maintain patient deidentification, incident case counts of 10 or fewer are censored and displayed as 10. Propensity score matching was implemented while controlling for baseline demographic variables (index age, sex, and race), body mass index, socioeconomic and lifestyle characteristics (socioeconomic or psychosocial–related health hazards, Family history of other mental and behavioral disorders (Z81.8), and mental or behavioral disorders associated with psychoactive substance use), healthcare utilization patterns (ambulatory and inpatient encounters), and a set of prespecified comorbidities, including essential hypertension, diabetes mellitus chronic ischemic heart disease, cerebral infarction, chronic kidney disease, and vitamin D deficiency.

**Table S10. Sensitivity analysis: applying 12-months of lagging period after index date**

|  | Comparing with Ezetimibe users | | | Comparing with Fenofibrates users | | | Comparing with Cholestyramine users | | |
| --- | --- | --- | --- | --- | --- | --- | --- | --- | --- |
| **Outcomes** | PCSK9 inhibitors cohort (%)^a^ | Ezetimibe cohort (%) | HR (95% CI)^a^ | PCSK9 inhibitors cohort (%)^a^ | Fenofibrates cohort (%) | HR (95% CI)^a^ | PCSK9 inhibitors cohort (%)^a^ | Cholestyramine cohort (%) | HR (95% CI)^a^ |
| All-caused dementia | 295 (0.8) | 545 (1.5) | 0.648 (0.562,0.746) | 334 (0.8) | 1076 (2.5) | 0.429 (0.379,0.485) | 226 (1.0) | 659 (2.9) | 0.447 (0.384,0.52) |
| Vascular dementia | 74 (0.2) | 127 (0.4) | 0.705 (0.529,0.939) | 89 (0.2) | 219 (0.5) | 0.561 (0.438,0.719) | 53 (0.2) | 116 (0.5) | 0.598 (0.432,0.828) |
| Parkinson’s disease | 127 (0.4) | 161 (0.5) | 0.939 (0.744,1.185) | 164 (0.4) | 233 (0.5) | 0.971 (0.794,1.187) | 84 (0.4) | 176 (0.8) | 0.623 (0.48,0.808) |
| Alzheimer’s disease | 97 (0.3) | 171 (0.5) | 0.69 (0.538,0.886) | 98 (0.2) | 274 (0.6) | 0.5 (0.396,0.63) | 60 (0.3) | 173 (0.8) | 0.462 (0.344,0.62) |
| Other degenerative diseases of nervous system | 410 (1.2) | 544 (1.5) | 0.902 (0.793,1.026) | 478 (1.1) | 829 (1.9) | 0.797 (0.712,0.892) | 306 (1.3) | 612 (2.7) | 0.651 (0.568,0.747) |

PCSK9 inhibitors, Proprotein Convertase Subtilisin/Kexin Type 9 Inhibitor; In the TriNetX analytics platform, to maintain patient deidentification, incident case counts of 10 or fewer are censored and displayed as 10. Propensity score matching was implemented while controlling for baseline demographic variables (index age, sex, and race), body mass index, socioeconomic and lifestyle characteristics (socioeconomic or psychosocial–related health hazards, Family history of other mental and behavioral disorders (Z81.8), and mental or behavioral disorders associated with psychoactive substance use), healthcare utilization patterns (ambulatory and inpatient encounters), and a set of prespecified comorbidities, including essential hypertension, diabetes mellitus chronic ischemic heart disease, cerebral infarction, chronic kidney disease, and vitamin D deficiency.

**Table S11. Sensitivity analysis: applying 24-months of lagging period after index date**

|  | Comparing with Ezetimibe users | | | Comparing with Fenofibrates users | | | Comparing with Cholestyramine users | | |
| --- | --- | --- | --- | --- | --- | --- | --- | --- | --- |
| **Outcomes** | PCSK9 inhibitors cohort (%)^a^ | Ezetimibe cohort (%) | HR (95% CI)^a^ | PCSK9 inhibitors cohort (%)^a^ | Fenofibrates cohort (%) | HR (95% CI)^a^ | PCSK9 inhibitors cohort (%)^a^ | Cholestyramine cohort (%) | HR (95% CI)^a^ |
| All-caused dementia | 221 (0.6) | 428 (1.2) | 0.653 (0.555,0.768) | 266 (0.6) | 877 (2.0) | 0.468 (0.408,0.536) | 173 (0.8) | 506 (2.2) | 0.493 (0.415,0.586) |
| Vascular dementia | 55 (0.2) | 97 (0.3) | 0.722 (0.518,1.005) | 65 (0.2) | 169 (0.4) | 0.594 (0.446,0.792) | 33 (0.1) | 87 (0.4) | 0.554 (0.371,0.828) |
| Parkinson’s disease | 96 (0.3) | 130 (0.4) | 0.928 (0.713,1.208) | 125 (0.3) | 204 (0.5) | 0.928 (0.742,1.16) | 66 (0.3) | 143 (0.6) | 0.666 (0.497,0.892) |
| Alzheimer’s disease | 70 (0.2) | 143 (0.4) | 0.624 (0.469,0.831) | 78 (0.2) | 226 (0.5) | 0.53 (0.41,0.686) | 46 (0.2) | 132 (0.6) | 0.505 (0.361,0.707) |
| Other degenerative diseases of nervous system | 284 (0.8) | 404 (1.1) | 0.892 (0.766,1.038) | 342 (0.8) | 638 (1.5) | 0.829 (0.726,0.945) | 213 (0.9) | 439 (1.9) | 0.704 (0.597,0.829) |

PCSK9 inhibitors, Proprotein Convertase Subtilisin/Kexin Type 9 Inhibitor; In the TriNetX analytics platform, to maintain patient deidentification, incident case counts of 10 or fewer are censored and displayed as 10. Propensity score matching was implemented while controlling for baseline demographic variables (index age, sex, and race), body mass index, socioeconomic and lifestyle characteristics (socioeconomic or psychosocial–related health hazards, Family history of other mental and behavioral disorders (Z81.8), and mental or behavioral disorders associated with psychoactive substance use), healthcare utilization patterns (ambulatory and inpatient encounters), and a set of prespecified comorbidities, including essential hypertension, diabetes mellitus chronic ischemic heart disease, cerebral infarction, chronic kidney disease, and vitamin D deficiency.

**Table S12. Sensitivity analysis: applying 1-year of follow-up period after index date**

|  | Comparing with Ezetimibe users | | | Comparing with Fenofibrates users | | | Comparing with Cholestyramine users | | |
| --- | --- | --- | --- | --- | --- | --- | --- | --- | --- |
| **Outcomes** | PCSK9 inhibitors cohort (%)^a^ | Ezetimibe cohort (%) | HR (95% CI)^a^ | PCSK9 inhibitors cohort (%)^a^ | Fenofibrates cohort (%) | HR (95% CI)^a^ | PCSK9 inhibitors cohort (%)^a^ | Cholestyramine cohort (%) | HR (95% CI)^a^ |
| All-caused dementia | 130 (0.4) | 197 (0.6) | 0.664 (0.532,0.828) | 142 (0.3) | 381 (0.9) | 0.372 (0.307,0.451) | 86 (0.4) | 285 (1.2) | 0.299 (0.235,0.38) |
| Vascular dementia | 25 (<0.1) | 34 (<0.1) | 0.741 (0.442,1.241) | 36 (<0.1) | 69 (0.2) | 0.523 (0.349,0.782) | 16 (<0.1) | 46 (0.2) | 0.346 (0.196,0.61) |
| Parkinson’s disease | 55 (0.2) | 51 (0.1) | 1.085 (0.741,1.588) | 66 (0.2) | 89 (0.2) | 0.742 (0.54,1.021) | 30 (0.1) | 70 (0.3) | 0.425 (0.277,0.652) |
| Alzheimer’s disease | 34 (<0.1) | 36 (<0.1) | 0.952 (0.596,1.522) | 36 (<0.1) | 74 (0.2) | 0.487 (0.327,0.726) | 24 (0.1) | 65 (0.3) | 0.367 (0.23,0.587) |
| Other degenerative diseases of nervous system | 170 (0.5) | 214 (0.6) | 0.801 (0.655,0.979) | 184 (0.4) | 282 (0.7) | 0.653 (0.542,0.786) | 112 (0.5) | 231 (1.0) | 0.481 (0.384,0.602) |

PCSK9 inhibitors, Proprotein Convertase Subtilisin/Kexin Type 9 Inhibitor; In the TriNetX analytics platform, to maintain patient deidentification, incident case counts of 10 or fewer are censored and displayed as 10. Propensity score matching was implemented while controlling for baseline demographic variables (index age, sex, and race), body mass index, socioeconomic and lifestyle characteristics (socioeconomic or psychosocial–related health hazards, Family history of other mental and behavioral disorders (Z81.8), and mental or behavioral disorders associated with psychoactive substance use), healthcare utilization patterns (ambulatory and inpatient encounters), and a set of prespecified comorbidities, including essential hypertension, diabetes mellitus chronic ischemic heart disease, cerebral infarction, chronic kidney disease, and vitamin D deficiency.

**Table S13. Sensitivity analysis: applying 2-year of follow-up period after index date**

|  | Comparing with Ezetimibe users | | | Comparing with Fenofibrates users | | | Comparing with Cholestyramine users | | |
| --- | --- | --- | --- | --- | --- | --- | --- | --- | --- |
| **Outcomes** | PCSK9 inhibitors cohort (%)^a^ | Ezetimibe cohort (%) | HR (95% CI)^a^ | PCSK9 inhibitors cohort (%)^a^ | Fenofibrates cohort (%) | HR (95% CI)^a^ | PCSK9 inhibitors cohort (%)^a^ | Cholestyramine cohort (%) | HR (95% CI)^a^ |
| All-caused dementia | 216 (0.6) | 348 (1.0) | 0.646 (0.545,0.765) | 226 (0.5) | 646 (1.5) | 0.368 (0.316,0.428) | 151 (0.7) | 470 (2.1) | 0.332 (0.277,0.399) |
| Vascular dementia | 47 (0.1) | 71 (0.2) | 0.696 (0.481,1.006) | 63 (0.1) | 129 (0.3) | 0.52 (0.384,0.703) | 38 (0.2) | 82 (0.4) | 0.489 (0.333,0.718) |
| Parkinson’s disease | 86 (0.2) | 97 (0.3) | 0.925 (0.692,1.237) | 105 (0.2) | 136 (0.3) | 0.811 (0.628,1.046) | 52 (0.2) | 113 (0.5) | 0.478 (0.344,0.664) |
| Alzheimer’s disease | 63 (0.2) | 76 (0.2) | 0.871 (0.624,1.216) | 58 (0.1) | 144 (0.3) | 0.428 (0.316,0.581) | 37 (0.2) | 111 (0.5) | 0.348 (0.24,0.506) |
| Other degenerative diseases of nervous system | 309 (0.9) | 366 (1.0) | 0.88 (0.757,1.024) | 334 (0.8) | 509 (1.2) | 0.696 (0.606,0.799) | 216 (0.9) | 429 (1.9) | 0.526 (0.446,0.619) |

PCSK9 inhibitors, Proprotein Convertase Subtilisin/Kexin Type 9 Inhibitor; In the TriNetX analytics platform, to maintain patient deidentification, incident case counts of 10 or fewer are censored and displayed as 10. Propensity score matching was implemented while controlling for baseline demographic variables (index age, sex, and race), body mass index, socioeconomic and lifestyle characteristics (socioeconomic or psychosocial–related health hazards, Family history of other mental and behavioral disorders (Z81.8), and mental or behavioral disorders associated with psychoactive substance use), healthcare utilization patterns (ambulatory and inpatient encounters), and a set of prespecified comorbidities, including essential hypertension, diabetes mellitus chronic ischemic heart disease, cerebral infarction, chronic kidney disease, and vitamin D deficiency.

**Table S14. Sensitivity analysis: applying 3-year of follow-up period after index date**

|  | Comparing with Ezetimibe users | | | Comparing with Fenofibrates users | | | Comparing with Cholestyramine users | | |
| --- | --- | --- | --- | --- | --- | --- | --- | --- | --- |
| **Outcomes** | PCSK9 inhibitors cohort (%)^a^ | Ezetimibe cohort (%) | HR (95% CI)^a^ | PCSK9 inhibitors cohort (%)^a^ | Fenofibrates cohort (%) | HR (95% CI)^a^ | PCSK9 inhibitors cohort (%)^a^ | Cholestyramine cohort (%) | HR (95% CI)^a^ |
| All-caused dementia | 283 (0.8) | 469 (1.3) | 0.65 (0.561,0.753) | 308 (0.7) | 875 (2.0) | 0.395 (0.347,0.45) | 205 (0.9) | 613 (2.7) | 0.365 (0.312,0.428) |
| Vascular dementia | 63 (0.2) | 106 (0.3) | 0.65 (0.476,0.889) | 85 (0.2) | 177 (0.4) | 0.546 (0.422,0.708) | 48 (0.2) | 106 (0.5) | 0.501 (0.356,0.705) |
| Parkinson’s disease | 116 (0.3) | 141 (0.4) | 0.893 (0.699,1.142) | 148 (0.3) | 189 (0.4) | 0.887 (0.715,1.101) | 73 (0.3) | 151 (0.7) | 0.535 (0.404,0.707) |
| Alzheimer’s disease | 83 (0.2) | 114 (0.3) | 0.795 (0.599,1.055) | 84 (0.2) | 219 (0.5) | 0.443 (0.344,0.57) | 54 (0.2) | 146 (0.6) | 0.41 (0.3,0.561) |
| Other degenerative diseases of nervous system | 409 (1.2) | 494 (1.4) | 0.895 (0.785,1.021) | 451 (1.0) | 723 (1.7) | 0.711 (0.632,0.8) | 294 (1.3) | 574 (2.5) | 0.567 (0.493,0.653) |

PCSK9 inhibitors, Proprotein Convertase Subtilisin/Kexin Type 9 Inhibitor; In the TriNetX analytics platform, to maintain patient deidentification, incident case counts of 10 or fewer are censored and displayed as 10. Propensity score matching was implemented while controlling for baseline demographic variables (index age, sex, and race), body mass index, socioeconomic and lifestyle characteristics (socioeconomic or psychosocial–related health hazards, Family history of other mental and behavioral disorders (Z81.8), and mental or behavioral disorders associated with psychoactive substance use), healthcare utilization patterns (ambulatory and inpatient encounters), and a set of prespecified comorbidities, including essential hypertension, diabetes mellitus chronic ischemic heart disease, cerebral infarction, chronic kidney disease, and vitamin D deficiency.

**Table S15. Sensitivity analysis: applying 4-year of follow-up period after index date**

|  | Comparing with Ezetimibe users | | | Comparing with Fenofibrates users | | | Comparing with Cholestyramine users | | |
| --- | --- | --- | --- | --- | --- | --- | --- | --- | --- |
| **Outcomes** | PCSK9 inhibitors cohort (%)^a^ | Ezetimibe cohort (%) | HR (95% CI)^a^ | PCSK9 inhibitors cohort (%)^a^ | Fenofibrates cohort (%) | HR (95% CI)^a^ | PCSK9 inhibitors cohort (%)^a^ | Cholestyramine cohort (%) | HR (95% CI)^a^ |
| All-caused dementia | 336 (0.9) | 561 (1.6) | 0.665 (0.581,0.761) | 371 (0.9) | 1077 (2.5) | 0.412 (0.366,0.464) | 250 (1.1) | 747 (3.3) | 0.387 (0.335,0.447) |
| Vascular dementia | 70 (0.2) | 129 (0.4) | 0.609 (0.455,0.816) | 95 (0.2) | 223 (0.5) | 0.515 (0.405,0.655) | 56 (0.2) | 135 (0.6) | 0.488 (0.357,0.668) |
| Parkinson’s disease | 133 (0.4) | 165 (0.5) | 0.899 (0.715,1.13) | 167 (0.4) | 237 (0.6) | 0.848 (0.695,1.035) | 86 (0.4) | 183 (0.8) | 0.549 (0.424,0.71) |
| Alzheimer’s disease | 107 (0.3) | 147 (0.4) | 0.827 (0.644,1.061) | 106 (0.2) | 275 (0.6) | 0.477 (0.381,0.597) | 71 (0.3) | 184 (0.8) | 0.458 (0.348,0.603) |
| Other degenerative diseases of nervous system | 486 (1.4) | 591 (1.7) | 0.917 (0.813,1.034) | 538 (1.2) | 888 (2.1) | 0.734 (0.66,0.818) | 345 (1.5) | 692 (3.0) | 0.581 (0.51,0.661) |

PCSK9 inhibitors, Proprotein Convertase Subtilisin/Kexin Type 9 Inhibitor; In the TriNetX analytics platform, to maintain patient deidentification, incident case counts of 10 or fewer are censored and displayed as 10. Propensity score matching was implemented while controlling for baseline demographic variables (index age, sex, and race), body mass index, socioeconomic and lifestyle characteristics (socioeconomic or psychosocial–related health hazards, Family history of other mental and behavioral disorders (Z81.8), and mental or behavioral disorders associated with psychoactive substance use), healthcare utilization patterns (ambulatory and inpatient encounters), and a set of prespecified comorbidities, including essential hypertension, diabetes mellitus chronic ischemic heart disease, cerebral infarction, chronic kidney disease, and vitamin D deficiency.

**Table S16. Sensitivity analysis: results of crude model prior to propensity score matching**

|  | Comparing with Ezetimibe users | | | Comparing with Fenofibrates users | | | Comparing with Cholestyramine users | | |
| --- | --- | --- | --- | --- | --- | --- | --- | --- | --- |
| **Outcomes** | PCSK9 inhibitors cohort (%)^a^ | Ezetimibe cohort (%) | HR (95% CI)^a^ | PCSK9 inhibitors cohort (%)^a^ | Fenofibrates cohort (%) | HR (95% CI)^a^ | PCSK9 inhibitors cohort (%)^a^ | Cholestyramine cohort (%) | HR (95% CI)^a^ |
| All-caused dementia | 380 (1.1) | 5638 (2.1) | 0.587 (0.529,0.651) | 532 (1.1) | 3986 (2.0) | 0.692 (0.632,0.758) | 570 (1.1) | 1044 (3.9) | 0.331 (0.298,0.366) |
| Vascular dementia | 87 (0.2) | 1160 (0.4) | 0.659 (0.53,0.819) | 137 (0.3) | 792 (0.4) | 0.91 (0.758,1.091) | 142 (0.3) | 181 (0.7) | 0.486 (0.39,0.606) |
| Parkinson’s disease | 152 (0.4) | 1508 (0.6) | 0.88 (0.745,1.04) | 217 (0.4) | 1000 (0.5) | 1.13 (0.975,1.31) | 225 (0.4) | 233 (0.9) | 0.596 (0.496,0.717) |
| Alzheimer’s disease | 122 (0.3) | 1574 (0.6) | 0.686 (0.571,0.825) | 158 (0.3) | 969 (0.5) | 0.863 (0.729,1.022) | 166 (0.3) | 252 (0.9) | 0.414 (0.34,0.504) |
| Other degenerative diseases of nervous system | 528 (1.5) | 4868 (1.8) | 0.943 (0.862,1.032) | 763 (1.5) | 3210 (1.6) | 1.245 (1.15,1.347) | 802 (1.5) | 929 (3.4) | 0.528 (0.48,0.58) |

PCSK9 inhibitors, Proprotein Convertase Subtilisin/Kexin Type 9 Inhibitor; In the TriNetX analytics platform, to maintain patient deidentification, incident case counts of 10 or fewer are censored and displayed as 10. Propensity score matching was not performed in this analysis.

**Table S17. Sensitivity analysis: results of propensity score matching model 1 (age and sex)**

|  | Comparing with Ezetimibe users | | | Comparing with Fenofibrates users | | | Comparing with Cholestyramine users | | |
| --- | --- | --- | --- | --- | --- | --- | --- | --- | --- |
| **Outcomes** | PCSK9 inhibitors cohort (%)^a^ | Ezetimibe cohort (%) | HR (95% CI)^a^ | PCSK9 inhibitors cohort (%)^a^ | Fenofibrates cohort (%) | HR (95% CI)^a^ | PCSK9 inhibitors cohort (%)^a^ | Cholestyramine cohort (%) | HR (95% CI)^a^ |
| All-caused dementia | 380 (1.1) | 677 (1.9) | 0.631 (0.556,0.715) | 178 (0.4) | 424 (0.9) | 0.417 (0.35,0.497) | 326 (1.2) | 953 (3.6) | 0.402 (0.354,0.456) |
| Vascular dementia | 87 (0.2) | 146 (0.4) | 0.676 (0.518,0.882) | 48 (0.1) | 75 (0.2) | 0.637 (0.444,0.915) | 74 (0.3) | 171 (0.7) | 0.518 (0.394,0.682) |
| Parkinson’s disease | 151 (0.4) | 184 (0.5) | 0.922 (0.743,1.143) | 71 (0.1) | 103 (0.2) | 0.686 (0.507,0.928) | 119 (0.5) | 227 (0.9) | 0.628 (0.503,0.785) |
| Alzheimer’s disease | 121 (0.3) | 194 (0.5) | 0.717 (0.571,0.9) | 44 (<0.1) | 81 (0.2) | 0.541 (0.375,0.781) | 100 (0.4) | 231 (0.9) | 0.531 (0.42,0.672) |
| Other degenerative diseases of nervous system | 527 (1.5) | 630 (1.8) | 0.937 (0.835,1.052) | 230 (0.5) | 310 (0.6) | 0.738 (0.622,0.875) | 446 (1.7) | 878 (3.4) | 0.604 (0.539,0.677) |

PCSK9 inhibitors, Proprotein Convertase Subtilisin/Kexin Type 9 Inhibitor; In the TriNetX analytics platform, to maintain patient deidentification, incident case counts of 10 or fewer are censored and displayed as 10. Propensity score matching was implemented while controlling for age and sex

**Table S18. Sensitivity analysis: results of propensity score matching model 2 (matching model + comedications)**

|  | Comparing with Ezetimibe users | | | Comparing with Fenofibrates users | | | Comparing with Cholestyramine users | | |
| --- | --- | --- | --- | --- | --- | --- | --- | --- | --- |
| **Outcomes** | PCSK9 inhibitors cohort (%)^a^ | Ezetimibe cohort (%) | HR (95% CI)^a^ | PCSK9 inhibitors cohort (%)^a^ | Fenofibrates cohort (%) | HR (95% CI)^a^ | PCSK9 inhibitors cohort (%)^a^ | Cholestyramine cohort (%) | HR (95% CI)^a^ |
| All-caused dementia | 380 (1.1) | 677 (1.9) | 0.631 (0.556,0.715) | 178 (0.4) | 424 (0.9) | 0.417 (0.35,0.497) | 326 (1.2) | 953 (3.6) | 0.402 (0.354,0.456) |
| Vascular dementia | 87 (0.2) | 146 (0.4) | 0.676 (0.518,0.882) | 48 (0.1) | 75 (0.2) | 0.637 (0.444,0.915) | 74 (0.3) | 171 (0.7) | 0.518 (0.394,0.682) |
| Parkinson’s disease | 151 (0.4) | 184 (0.5) | 0.922 (0.743,1.143) | 71 (0.1) | 103 (0.2) | 0.686 (0.507,0.928) | 119 (0.5) | 227 (0.9) | 0.628 (0.503,0.785) |
| Alzheimer’s disease | 121 (0.3) | 194 (0.5) | 0.717 (0.571,0.9) | 44 (<0.1) | 81 (0.2) | 0.541 (0.375,0.781) | 100 (0.4) | 231 (0.9) | 0.531 (0.42,0.672) |
| Other degenerative diseases of nervous system | 527 (1.5) | 630 (1.8) | 0.937 (0.835,1.052) | 230 (0.5) | 310 (0.6) | 0.738 (0.622,0.875) | 446 (1.7) | 878 (3.4) | 0.604 (0.539,0.677) |

PCSK9 inhibitors, Proprotein Convertase Subtilisin/Kexin Type 9 Inhibitor; In the TriNetX analytics platform, to maintain patient deidentification, incident case counts of 10 or fewer are censored and displayed as 10. Propensity score matching was implemented while controlling for baseline demographic variables (index age, sex, and race), body mass index, socioeconomic and lifestyle characteristics (socioeconomic or psychosocial–related health hazards, Family history of other mental and behavioral disorders (Z81.8), and mental or behavioral disorders associated with psychoactive substance use), healthcare utilization patterns (ambulatory and inpatient encounters), and a set of prespecified comorbidities, including essential hypertension, diabetes mellitus chronic ischemic heart disease, cerebral infarction, chronic kidney disease, and vitamin D deficiency and comedications (vasoprotectives, beta blockers, agents acting on the renin-angiotensin system, aspirin, corticosteroids, diuretics, calcium channel blockers, vitamins, immunosuppressants, niacin).

**Table S19. Sensitivity analysis: results of intention-to-treat analysis applying as-started designation**

|  | Comparing with Ezetimibe users | | | Comparing with Fenofibrates users | | | Comparing with Cholestyramine users | | |
| --- | --- | --- | --- | --- | --- | --- | --- | --- | --- |
| **Outcomes** | PCSK9 inhibitors cohort (%)^a^ | Ezetimibe cohort (%) | HR (95% CI)^a^ | PCSK9 inhibitors cohort (%)^a^ | Fenofibrates cohort (%) | HR (95% CI)^a^ | PCSK9 inhibitors cohort (%)^a^ | Cholestyramine cohort (%) | HR (95% CI)^a^ |
| All-caused dementia | 586 (1.1) | 859 (1.6) | 0.747 (0.672,0.83) | 482 (1.0) | 1335 (2.8) | 0.459 (0.414,0.51) | 30 (1.4) | 92 (4.3) | 0.388 (0.257,0.588) |
| Vascular dementia | 147 (0.3) | 181 (0.3) | 0.896 (0.721,1.114) | 122 (0.3) | 276 (0.6) | 0.571 (0.461,0.708) | NA | NA | NA |
| Parkinson’s disease | 228 (0.4) | 255 (0.5) | 0.982 (0.821,1.174) | 202 (0.4) | 302 (0.6) | 0.87 (0.727,1.041) | NA | NA | NA |
| Alzheimer’s disease | 176 (0.3) | 274 (0.5) | 0.71 (0.588,0.859) | 150 (0.3) | 339 (0.7) | 0.586 (0.483,0.711) | ≤10 (≤0.1) | 21 (1.0) | 0.632 (0.296,1.348) |
| Other degenerative diseases of nervous system | 827 (1.5) | 970 (1.8) | 0.931 (0.848,1.021) | 686 (1.5) | 1096 (2.3) | 0.802 (0.728,0.882) | 48 (2.2) | 87 (4.0) | 0.652 (0.458,0.929) |

PCSK9 inhibitors, Proprotein Convertase Subtilisin/Kexin Type 9 Inhibitor; In the TriNetX analytics platform, to maintain patient deidentification, incident case counts of 10 or fewer are censored and displayed as 10. Propensity score matching was implemented while controlling for baseline demographic variables (index age, sex, and race), body mass index, socioeconomic and lifestyle characteristics (socioeconomic or psychosocial–related health hazards, Family history of other mental and behavioral disorders (Z81.8), and mental or behavioral disorders associated with psychoactive substance use), healthcare utilization patterns (ambulatory and inpatient encounters), and a set of prespecified comorbidities, including essential hypertension, diabetes mellitus chronic ischemic heart disease, cerebral infarction, chronic kidney disease, and vitamin D deficiency.

**Table S20. Baseline characteristics in the statin-unrestricted PCSK9 inhibitor sensitivity analysis after propensity score matching**

In this sensitivity analysis, the PCSK9 inhibitor cohort had no exclusion criterion related to statin use before, on, or after the index date. The expanded propensity-score model included clinically defined lipid categories and baseline concomitant medications. Continuous laboratory values are descriptive and were not matched as continuous variables.

| **Characteristic** | **PCSK9 inhibitor users (n=54,387)** | **Statin users (n=54,387)** | **SMD** |
| --- | --- | --- | --- |
| Age at Index | 63.0 ± 11.3 | 63.2 ± 12.4 | 0.018 |
| White | 40929 (75.255%) | 41212 (75.775%) | 0.012 |
| Male | 28613 (52.61%) | 28514 (52.428%) | 0.004 |
| Female | 25689 (47.234%) | 25812 (47.46%) | 0.005 |
| Black or African American | 4972 (9.142%) | 5397 (9.923%) | 0.027 |
| Unknown Race | 4740 (8.715%) | 4134 (7.601%) | 0.041 |
| Asian | 1798 (3.306%) | 2223 (4.087%) | 0.041 |
| Other Race | 1577 (2.9%) | 1055 (1.94%) | 0.062 |
| American Indian or Alaska Native | 234 (0.43%) | 168 (0.309%) | 0.020 |
| Native Hawaiian or Other Pacific Islander | 137 (0.252%) | 198 (0.364%) | 0.020 |
| Unknown Gender | 85 (0.156%) | 61 (0.112%) | 0.012 |
| BMI | 30.4 ± 6.1 | 31.2 ± 6.9 | 0.120 |
| BMI, At least 25 kg/m2 | 36702 (67.483%) | 36376 (66.884%) | 0.013 |
| Triglyceride [Mass/volume] in Serum, Plasma or Blood | 177.6 ± 208.3 | 165.5 ± 151.4 | 0.067 |
| Triglyceride [Mass/volume] in Serum, Plasma or Blood, At least 200 mg/dL | 16332 (30.029%) | 15579 (28.645%) | 0.030 |
| Cholesterol in HDL [Mass/volume] in Serum or Plasma | 47.8 ± 16.9 | 47.8 ± 17.0 | 0.001 |
| Cholesterol in HDL [Mass/volume] in Serum or Plasma, At most 40 mg/dL | 16983 (31.226%) | 16307 (29.983%) | 0.027 |
| Cholesterol in LDL [Mass/volume] in Serum or Plasma | 127.9 ± 57.3 | 116.1 ± 45.4 | 0.228 |
| Cholesterol in LDL [Mass/volume] in Serum or Plasma, At least 130 mg/dL | 24424 (44.908%) | 23350 (42.933%) | 0.040 |
| Cholesterol [Mass/volume] in Serum or Plasma | 209.0 ± 66.9 | 194.7 ± 55.0 | 0.233 |
| Cholesterol [Mass/volume] in Serum or Plasma, At least 200 mg/dL | 26788 (49.254%) | 25546 (46.971%) | 0.046 |
| Hemoglobin A1c/Hemoglobin.total in Blood | 6.4 ± 1.5 | 6.5 ± 1.7 | 0.090 |
| Hemoglobin A1c/Hemoglobin.total in Blood, At least 5.7 % | 20621 (37.915%) | 20400 (37.509%) | 0.008 |
| Urate [Mass/volume] in Serum or Plasma | 6.0 ± 1.8 | 6.1 ± 2.0 | 0.073 |
| Urate [Mass/volume] in Serum or Plasma, At least 7.5 mg/dL | 1585 (2.914%) | 1978 (3.637%) | 0.041 |
| Essential (primary) hypertension | 36647 (67.382%) | 36179 (66.521%) | 0.018 |
| Chronic ischemic heart disease | 30646 (56.348%) | 31193 (57.354%) | 0.020 |
| Diabetes mellitus | 16376 (30.11%) | 15645 (28.766%) | 0.029 |
| Vitamin D deficiency | 9257 (17.021%) | 8795 (16.171%) | 0.023 |
| Mental and behavioral disorders due to psychoactive substance use | 8392 (15.43%) | 8034 (14.772%) | 0.018 |
| Chronic kidney disease (CKD) | 5920 (10.885%) | 5652 (10.392%) | 0.016 |
| Diseases of liver | 4747 (8.728%) | 3784 (6.958%) | 0.066 |
| Cerebral infarction | 3422 (6.292%) | 3213 (5.908%) | 0.016 |
| Family history of diabetes mellitus | 1764 (3.243%) | 1486 (2.732%) | 0.030 |
| Persons with potential health hazards related to socioeconomic and psychosocial circumstances | 1111 (2.043%) | 1034 (1.901%) | 0.010 |
| Psoriasis | 1010 (1.857%) | 927 (1.704%) | 0.012 |
| Family history of other endocrine, nutritional and metabolic diseases | 456 (0.838%) | 265 (0.487%) | 0.043 |
| Rheumatoid arthritis with rheumatoid factor | 349 (0.642%) | 359 (0.66%) | 0.002 |
| Systemic lupus erythematosus (SLE) | 323 (0.594%) | 321 (0.59%) | 0.000 |
| Human immunodeficiency virus [HIV] disease | 192 (0.353%) | 185 (0.34%) | 0.002 |
| Family history of other mental and behavioral disorders | 178 (0.327%) | 177 (0.325%) | 0.000 |
| Visit: Ambulatory | 45167 (83.047%) | 44846 (82.457%) | 0.016 |
| Visit: Inpatient Encounter | 18575 (34.153%) | 18041 (33.172%) | 0.021 |
| VASOPROTECTIVES | 33102 (60.864%) | 32583 (59.91%) | 0.020 |
| BETA BLOCKING AGENTS | 29438 (54.127%) | 29421 (54.096%) | 0.001 |
| AGENTS ACTING ON THE RENIN-ANGIOTENSIN SYSTEM | 27471 (50.51%) | 27289 (50.176%) | 0.007 |
| aspirin | 27406 (50.391%) | 27212 (50.034%) | 0.007 |
| CORTICOSTEROIDS FOR SYSTEMIC USE | 23828 (43.812%) | 23218 (42.69%) | 0.023 |
| DIURETICS | 19990 (36.755%) | 19855 (36.507%) | 0.005 |
| CALCIUM CHANNEL BLOCKERS | 19935 (36.654%) | 19628 (36.09%) | 0.012 |
| VITAMINS | 17317 (31.84%) | 16995 (31.248%) | 0.013 |
| IMMUNOSUPPRESSANTS | 2398 (4.409%) | 1897 (3.488%) | 0.047 |
| Varicella zoster vaccines | 2205 (4.054%) | 2539 (4.668%) | 0.030 |
| niacin | 1890 (3.475%) | 1846 (3.394%) | 0.004 |

Values are n (%) or mean ± SD. Laboratory values were not imputed. All categorical lipid indicators and concomitant medications entered in the expanded model had post-matching SMDs below 0.10; continuous descriptive LDL-C, total cholesterol, and BMI measurements retained residual imbalance.

**Table S21. Outcomes in the statin-unrestricted PCSK9 inhibitor sensitivity analysis**

| **Outcome** | **PCSK9 inhibitor users, events** | **Statin users, events** | **Hazard ratio (95% CI)** |
| --- | --- | --- | --- |
| All-cause dementia | 659 | 1,486 | 0.529 (0.482–0.580) |
| Vascular dementia | 151 | 323 | 0.561 (0.462–0.681) |
| Alzheimer’s disease | 199 | 417 | 0.585 (0.494–0.693) |
| Parkinson’s disease | 242 | 302 | 0.957 (0.808–1.135) |
| Other degenerative diseases of the nervous system | 938 | 1,400 | 0.806 (0.742–0.876) |

Matched cohort size: 54,387 per group. PCSK9 inhibitor users were not excluded on the basis of statin exposure before, on, or after the index date.

**Table S22. Sensitivity analyses retaining patients with a recorded death**

| **Framework** | **Outcome** | **PCSK9 inhibitor users, events** | **Statin users, events** | **Hazard ratio (95% CI)** |
| --- | --- | --- | --- | --- |
| Restricted exposure (n=28,665/group) | All-cause dementia | 391 | 1,067 | 0.445 (0.396–0.500) |
| Restricted exposure | Vascular dementia | 83 | 228 | 0.442 (0.343–0.569) |
| Restricted exposure | Alzheimer’s disease | 123 | 278 | 0.544 (0.439–0.673) |
| Restricted exposure | Parkinson’s disease | 137 | 195 | 0.862 (0.692–1.075) |
| Restricted exposure | Other degenerative diseases of the nervous system | 500 | 848 | 0.713 (0.638–0.796) |
| As-started (n=53,734/group) | All-cause dementia | 852 | 1,800 | 0.544 (0.501–0.590) |
| As-started | Vascular dementia | 193 | 371 | 0.604 (0.507–0.720) |
| As-started | Alzheimer’s disease | 241 | 441 | 0.644 (0.550–0.754) |
| As-started | Parkinson’s disease | 281 | 376 | 0.854 (0.731–0.998) |
| As-started | Other degenerative diseases of the nervous system | 1,055 | 1,558 | 0.781 (0.722–0.845) |

These analyses retained patients with a recorded death. It does not constitute a formal cause-specific or Fine-Gray competing-risk analysis.

**Table S23. Stratification analysis: male population**

|  | Comparing with Ezetimibe users | | | Comparing with Fenofibrates users | | | Comparing with Cholestyramine users | | |
| --- | --- | --- | --- | --- | --- | --- | --- | --- | --- |
| **Outcomes** | PCSK9 inhibitors cohort (%)^a^ | Ezetimibe cohort (%) | HR (95% CI)^a^ | PCSK9 inhibitors cohort (%)^a^ | Fenofibrates cohort (%) | HR (95% CI)^a^ | PCSK9 inhibitors cohort (%)^a^ | Cholestyramine cohort (%) | HR (95% CI)^a^ |
| All-caused dementia | 173 (1.0) | 242 (1.3) | 0.805 (0.662,0.979) | 212 (0.9) | 526 (2.3) | 0.503 (0.429,0.591) | 103 (1.3) | 267 (3.4) | 0.444 (0.353,0.558) |
| Vascular dementia | 40 (0.2) | 59 (0.3) | 0.773 (0.517,1.155) | 57 (0.2) | 121 (0.5) | 0.596 (0.435,0.818) | 34 (0.4) | 53 (0.7) | 0.757 (0.491,1.166) |
| Parkinson’s disease | 98 (0.5) | 99 (0.5) | 1.13 (0.854,1.495) | 125 (0.5) | 159 (0.7) | 1.021 (0.807,1.293) | 55 (0.7) | 85 (1.1) | 0.777 (0.553,1.093) |
| Alzheimer’s disease | 52 (0.3) | 63 (0.3) | 0.937 (0.649,1.354) | 63 (0.3) | 121 (0.5) | 0.664 (0.489,0.902) | 31 (0.4) | 47 (0.6) | 0.756 (0.479,1.191) |
| Other degenerative diseases of nervous system | 247 (1.4) | 285 (1.6) | 0.963 (0.812,1.142) | 312 (1.4) | 463 (2.0) | 0.848 (0.734,0.98) | 141 (1.8) | 229 (2.9) | 0.722 (0.585,0.891) |

PCSK9 inhibitors, Proprotein Convertase Subtilisin/Kexin Type 9 Inhibitor; In the TriNetX analytics platform, to maintain patient deidentification, incident case counts of 10 or fewer are censored and displayed as 10. Propensity score matching was implemented while controlling for baseline demographic variables (index age, sex, and race), body mass index, socioeconomic and lifestyle characteristics (socioeconomic or psychosocial–related health hazards, Family history of other mental and behavioral disorders (Z81.8), and mental or behavioral disorders associated with psychoactive substance use), healthcare utilization patterns (ambulatory and inpatient encounters), and a set of prespecified comorbidities, including essential hypertension, diabetes mellitus chronic ischemic heart disease, cerebral infarction, chronic kidney disease, and vitamin D deficiency.

**Table S24. Stratification analysis: female population**

|  | Comparing with Ezetimibe users | | | Comparing with Fenofibrates users | | | Comparing with Cholestyramine users | | |
| --- | --- | --- | --- | --- | --- | --- | --- | --- | --- |
| **Outcomes** | PCSK9 inhibitors cohort (%)^a^ | Ezetimibe cohort (%) | HR (95% CI)^a^ | PCSK9 inhibitors cohort (%)^a^ | Fenofibrates cohort (%) | HR (95% CI)^a^ | PCSK9 inhibitors cohort (%)^a^ | Cholestyramine cohort (%) | HR (95% CI)^a^ |
| All-caused dementia | 205 (1.2) | 376 (2.2) | 0.638 (0.538,0.756) | 221 (1.1) | 729 (3.6) | 0.395 (0.339,0.459) | 178 (1.2) | 551 (3.7) | 0.402 (0.339,0.476) |
| Vascular dementia | 47 (0.3) | 77 (0.4) | 0.724 (0.503,1.041) | 54 (0.3) | 144 (0.7) | 0.506 (0.37,0.694) | 36 (0.2) | 98 (0.7) | 0.464 (0.316,0.681) |
| Parkinson’s disease | 53 (0.3) | 82 (0.5) | 0.752 (0.532,1.064) | 62 (0.3) | 106 (0.5) | 0.741 (0.541,1.016) | 53 (0.4) | 114 (0.8) | 0.573 (0.413,0.796) |
| Alzheimer’s disease | 69 (0.4) | 111 (0.6) | 0.747 (0.552,1.009) | 67 (0.3) | 195 (1.0) | 0.476 (0.36,0.629) | 49 (0.3) | 156 (1.0) | 0.404 (0.292,0.557) |
| Other degenerative diseases of nervous system | 278 (1.6) | 368 (2.1) | 0.878 (0.751,1.026) | 302 (1.5) | 583 (2.9) | 0.684 (0.595,0.787) | 240 (1.6) | 530 (3.5) | 0.559 (0.479,0.651) |

PCSK9 inhibitors, Proprotein Convertase Subtilisin/Kexin Type 9 Inhibitor; In the TriNetX analytics platform, to maintain patient deidentification, incident case counts of 10 or fewer are censored and displayed as 10. Propensity score matching was implemented while controlling for baseline demographic variables (index age, sex, and race), body mass index, socioeconomic and lifestyle characteristics (socioeconomic or psychosocial–related health hazards, Family history of other mental and behavioral disorders (Z81.8), and mental or behavioral disorders associated with psychoactive substance use), healthcare utilization patterns (ambulatory and inpatient encounters), and a set of prespecified comorbidities, including essential hypertension, diabetes mellitus chronic ischemic heart disease, cerebral infarction, chronic kidney disease, and vitamin D deficiency.

**Table S25. Stratification analysis: 50-64 years old population**

|  | Comparing with Ezetimibe users | | | Comparing with Fenofibrates users | | | Comparing with Cholestyramine users | | |
| --- | --- | --- | --- | --- | --- | --- | --- | --- | --- |
| **Outcomes** | PCSK9 inhibitors cohort (%)^a^ | Ezetimibe cohort (%) | HR (95% CI)^a^ | PCSK9 inhibitors cohort (%)^a^ | Fenofibrates cohort (%) | HR (95% CI)^a^ | PCSK9 inhibitors cohort (%)^a^ | Cholestyramine cohort (%) | HR (95% CI)^a^ |
| All-caused dementia | 27 (0.2) | 41 (0.4) | 0.718 (0.442,1.168) | 29 (0.2) | 84 (0.6) | 0.409 (0.268,0.625) | 18 (0.3) | 45 (0.7) | 0.468 (0.271,0.81) |
| Vascular dementia | NA | NA | NA | NA | NA | NA | NA | NA | NA |
| Parkinson’s disease | 20 (0.2) | 14 (0.1) | 1.541 (0.778,3.052) | 29 (0.2) | 35 (0.3) | 1.006 (0.614,1.649) | NA | NA | NA |
| Alzheimer’s disease | NA | NA | NA | NA | NA | NA | NA | NA | NA |
| Other degenerative diseases of nervous system | 52 (0.4) | 70 (0.6) | 0.817 (0.571,1.17) | 70 (0.5) | 109 (0.8) | 0.764 (0.565,1.032) | 35 (0.6) | 81 (1.3) | 0.507 (0.341,0.755) |

PCSK9 inhibitors, Proprotein Convertase Subtilisin/Kexin Type 9 Inhibitor; In the TriNetX analytics platform, to maintain patient deidentification, incident case counts of 10 or fewer are censored and displayed as 10. Propensity score matching was implemented while controlling for baseline demographic variables (index age, sex, and race), body mass index, socioeconomic and lifestyle characteristics (socioeconomic or psychosocial–related health hazards, Family history of other mental and behavioral disorders (Z81.8), and mental or behavioral disorders associated with psychoactive substance use), healthcare utilization patterns (ambulatory and inpatient encounters), and a set of prespecified comorbidities, including essential hypertension, diabetes mellitus chronic ischemic heart disease, cerebral infarction, chronic kidney disease, and vitamin D deficiency.

**Table S26. Stratification analysis: greater than 65 years old population**

|  | Comparing with Ezetimibe users | | | Comparing with Fenofibrates users | | | Comparing with Cholestyramine users | | |
| --- | --- | --- | --- | --- | --- | --- | --- | --- | --- |
| **Outcomes** | PCSK9 inhibitors cohort (%)^a^ | Ezetimibe cohort (%) | HR (95% CI)^a^ | PCSK9 inhibitors cohort (%)^a^ | Fenofibrates cohort (%) | HR (95% CI)^a^ | PCSK9 inhibitors cohort (%)^a^ | Cholestyramine cohort (%) | HR (95% CI)^a^ |
| All-caused dementia | 353 (1.5) | 560 (2.4) | 0.709 (0.62,0.81) | 415 (1.4) | 1140 (3.9) | 0.454 (0.405,0.508) | 267 (1.7) | 737 (4.7) | 0.425 (0.369,0.489) |
| Vascular dementia | 81 (0.3) | 105 (0.4) | 0.886 (0.663,1.184) | 101 (0.3) | 222 (0.8) | 0.581 (0.458,0.736) | 61 (0.4) | 139 (0.9) | 0.521 (0.385,0.705) |
| Parkinson’s disease | 131 (0.6) | 166 (0.7) | 0.884 (0.703,1.112) | 164 (0.6) | 232 (0.8) | 0.9 (0.736,1.1) | 90 (0.6) | 180 (1.1) | 0.597 (0.463,0.77) |
| Alzheimer’s disease | 117 (0.5) | 172 (0.7) | 0.776 (0.613,0.982) | 133 (0.5) | 305 (1.1) | 0.562 (0.458,0.69) | 81 (0.5) | 192 (1.2) | 0.512 (0.395,0.665) |
| Other degenerative diseases of nervous system | 475 (2.0) | 557 (2.3) | 0.956 (0.845,1.08) | 583 (2.0) | 886 (3.1) | 0.828 (0.745,0.92) | 343 (2.2) | 636 (4.0) | 0.639 (0.56,0.729) |

PCSK9 inhibitors, Proprotein Convertase Subtilisin/Kexin Type 9 Inhibitor; In the TriNetX analytics platform, to maintain patient deidentification, incident case counts of 10 or fewer are censored and displayed as 10. Propensity score matching was implemented while controlling for baseline demographic variables (index age, sex, and race), body mass index, socioeconomic and lifestyle characteristics (socioeconomic or psychosocial–related health hazards, Family history of other mental and behavioral disorders (Z81.8), and mental or behavioral disorders associated with psychoactive substance use), healthcare utilization patterns (ambulatory and inpatient encounters), and a set of prespecified comorbidities, including essential hypertension, diabetes mellitus chronic ischemic heart disease, cerebral infarction, chronic kidney disease, and vitamin D deficiency.

**Table S27. Stratification analysis: White population**

|  | Comparing with Ezetimibe users | | | Comparing with Fenofibrates users | | | Comparing with Cholestyramine users | | |
| --- | --- | --- | --- | --- | --- | --- | --- | --- | --- |
| **Outcomes** | PCSK9 inhibitors cohort (%)^a^ | Ezetimibe cohort (%) | HR (95% CI)^a^ | PCSK9 inhibitors cohort (%)^a^ | Fenofibrates cohort (%) | HR (95% CI)^a^ | PCSK9 inhibitors cohort (%)^a^ | Cholestyramine cohort (%) | HR (95% CI)^a^ |
| All-caused dementia | 309 (1.1) | 452 (1.6) | 0.784 (0.679,0.907) | 339 (1.0) | 1035 (3.1) | 0.419 (0.37,0.474) | 233 (1.3) | 692 (3.7) | 0.403 (0.348,0.468) |
| Vascular dementia | 67 (0.2) | 88 (0.3) | 0.897 (0.652,1.233) | 81 (0.2) | 211 (0.6) | 0.501 (0.387,0.649) | 59 (0.3) | 115 (0.6) | 0.625 (0.456,0.857) |
| Parkinson’s disease | 136 (0.5) | 145 (0.5) | 1.085 (0.858,1.371) | 164 (0.5) | 242 (0.7) | 0.862 (0.706,1.053) | 96 (0.5) | 189 (1.0) | 0.619 (0.484,0.792) |
| Alzheimer’s disease | 102 (0.4) | 137 (0.5) | 0.857 (0.663,1.108) | 101 (0.3) | 262 (0.8) | 0.511 (0.406,0.644) | 68 (0.4) | 173 (0.9) | 0.486 (0.367,0.644) |
| Other degenerative diseases of nervous system | 437 (1.6) | 502 (1.8) | 0.988 (0.869,1.124) | 484 (1.4) | 858 (2.6) | 0.723 (0.647,0.809) | 303 (1.6) | 623 (3.3) | 0.591 (0.515,0.679) |

PCSK9 inhibitors, Proprotein Convertase Subtilisin/Kexin Type 9 Inhibitor; In the TriNetX analytics platform, to maintain patient deidentification, incident case counts of 10 or fewer are censored and displayed as 10. Propensity score matching was implemented while controlling for baseline demographic variables (index age, sex, and race), body mass index, socioeconomic and lifestyle characteristics (socioeconomic or psychosocial–related health hazards, Family history of other mental and behavioral disorders (Z81.8), and mental or behavioral disorders associated with psychoactive substance use), healthcare utilization patterns (ambulatory and inpatient encounters), and a set of prespecified comorbidities, including essential hypertension, diabetes mellitus chronic ischemic heart disease, cerebral infarction, chronic kidney disease, and vitamin D deficiency.

**Table S28. Stratification analysis: Black population**

|  | Comparing with Ezetimibe users | | | Comparing with Fenofibrates users | | | Comparing with Cholestyramine users | | |
| --- | --- | --- | --- | --- | --- | --- | --- | --- | --- |
| **Outcomes** | PCSK9 inhibitors cohort (%)^a^ | Ezetimibe cohort (%) | HR (95% CI)^a^ | PCSK9 inhibitors cohort (%)^a^ | Fenofibrates cohort (%) | HR (95% CI)^a^ | PCSK9 inhibitors cohort (%)^a^ | Cholestyramine cohort (%) | HR (95% CI)^a^ |
| All-caused dementia | 32 (1.1) | 71 (2.4) | 0.518 (0.341,0.787) | 43 (1.2) | 106 (2.9) | 0.534 (0.374,0.763) | 36 (1.7) | 89 (4.2) | 0.485 (0.329,0.716) |
| Vascular dementia | ≤10 (≤0.1) | 17 (0.6) | 0.653 (0.299,1.428) | 15 (0.4) | 27 (0.7) | 0.716 (0.379,1.353) | 12 (0.6) | 20 (1.0) | 0.725 (0.353,1.488) |
| Parkinson’s disease | NA | NA | NA | NA | NA | NA | NA | NA | NA |
| Alzheimer’s disease | NA | NA | NA | NA | NA | NA | NA | NA | NA |
| Other degenerative diseases of nervous system | 43 (1.4) | 55 (1.8) | 0.892 (0.598,1.33) | 57 (1.6) | 84 (2.3) | 0.922 (0.657,1.294) | 46 (2.2) | 83 (3.9) | 0.655 (0.456,0.94) |

PCSK9 inhibitors, Proprotein Convertase Subtilisin/Kexin Type 9 Inhibitor; In the TriNetX analytics platform, to maintain patient deidentification, incident case counts of 10 or fewer are censored and displayed as 10. Propensity score matching was implemented while controlling for baseline demographic variables (index age, sex, and race), body mass index, socioeconomic and lifestyle characteristics (socioeconomic or psychosocial–related health hazards, Family history of other mental and behavioral disorders (Z81.8), and mental or behavioral disorders associated with psychoactive substance use), healthcare utilization patterns (ambulatory and inpatient encounters), and a set of prespecified comorbidities, including essential hypertension, diabetes mellitus chronic ischemic heart disease, cerebral infarction, chronic kidney disease, and vitamin D deficiency.

**Table S29. Stratification analysis: Asian population**

|  | Comparing with Ezetimibe users | | | Comparing with Fenofibrates users | | | Comparing with Cholestyramine users | | |
| --- | --- | --- | --- | --- | --- | --- | --- | --- | --- |
| **Outcomes** | PCSK9 inhibitors cohort (%)^a^ | Ezetimibe cohort (%) | HR (95% CI)^a^ | PCSK9 inhibitors cohort (%)^a^ | Fenofibrates cohort (%) | HR (95% CI)^a^ | PCSK9 inhibitors cohort (%)^a^ | Cholestyramine cohort (%) | HR (95% CI)^a^ |
| All-caused dementia | 16 (1.7) | 18 (1.9) | 0.999 (0.509,1.961) | 16 (1.2) | 30 (2.2) | 0.705 (0.383,1.297) | NA | NA | NA |
| Vascular dementia | NA | NA | NA | NA | NA | NA | NA | NA | NA |
| Parkinson’s disease | NA | NA | NA | NA | NA | NA | NA | NA | NA |
| Alzheimer’s disease | NA | NA | NA | NA | NA | NA | NA | NA | NA |
| Other degenerative diseases of nervous system | 17 (1.8) | 16 (1.7) | 1.22 (0.616,2.417) | 24 (1.8) | 31 (2.3) | 1.039 (0.607,1.776) | NA | NA | NA |

PCSK9 inhibitors, Proprotein Convertase Subtilisin/Kexin Type 9 Inhibitor; In the TriNetX analytics platform, to maintain patient deidentification, incident case counts of 10 or fewer are censored and displayed as 10. Propensity score matching was implemented while controlling for baseline demographic variables (index age, sex, and race), body mass index, socioeconomic and lifestyle characteristics (socioeconomic or psychosocial–related health hazards, Family history of other mental and behavioral disorders (Z81.8), and mental or behavioral disorders associated with psychoactive substance use), healthcare utilization patterns (ambulatory and inpatient encounters), and a set of prespecified comorbidities, including essential hypertension, diabetes mellitus chronic ischemic heart disease, cerebral infarction, chronic kidney disease, and vitamin D deficiency.

**Table S30. Stratification analysis: population with LDL-C greater than 160 mg/dl**

|  | Comparing with Ezetimibe users | | | Comparing with Fenofibrates users | | | Comparing with Cholestyramine users | | |
| --- | --- | --- | --- | --- | --- | --- | --- | --- | --- |
| **Outcomes** | PCSK9 inhibitors cohort (%)^a^ | Ezetimibe cohort (%) | HR (95% CI)^a^ | PCSK9 inhibitors cohort (%)^a^ | Fenofibrates cohort (%) | HR (95% CI)^a^ | PCSK9 inhibitors cohort (%)^a^ | Cholestyramine cohort (%) | HR (95% CI)^a^ |
| All-caused dementia | 127 (1.0) | 232 (1.8) | 0.592 (0.477,0.735) | 100 (0.9) | 283 (2.5) | 0.423 (0.336,0.531) | 34 (1.0) | 92 (2.6) | 0.439 (0.296,0.651) |
| Vascular dementia | 32 (0.2) | 57 (0.4) | 0.609 (0.395,0.939) | 27 (0.2) | 62 (0.6) | 0.537 (0.341,0.845) | NA | NA | NA |
| Parkinson’s disease | 36 (0.3) | 44 (0.3) | 0.886 (0.57,1.377) | 36 (0.3) | 37 (0.3) | 1.176 (0.741,1.864) | NA | NA | NA |
| Alzheimer’s disease | 30 (0.2) | 73 (0.6) | 0.459 (0.3,0.702) | 21 (0.2) | 81 (0.7) | 0.313 (0.194,0.507) | NA | NA | NA |
| Other degenerative diseases of nervous system | 205 (1.6) | 265 (2.0) | 0.837 (0.697,1.004) | 157 (1.4) | 269 (2.4) | 0.707 (0.58,0.861) | 52 (1.5) | 106 (3.0) | 0.573 (0.411,0.8) |

PCSK9 inhibitors, Proprotein Convertase Subtilisin/Kexin Type 9 Inhibitor; In the TriNetX analytics platform, to maintain patient deidentification, incident case counts of 10 or fewer are censored and displayed as 10. Propensity score matching was implemented while controlling for baseline demographic variables (index age, sex, and race), body mass index, socioeconomic and lifestyle characteristics (socioeconomic or psychosocial–related health hazards, Family history of other mental and behavioral disorders (Z81.8), and mental or behavioral disorders associated with psychoactive substance use), healthcare utilization patterns (ambulatory and inpatient encounters), and a set of prespecified comorbidities, including essential hypertension, diabetes mellitus chronic ischemic heart disease, cerebral infarction, chronic kidney disease, and vitamin D deficiency.

**Table S31. Stratification analysis: population with LDL-C between 130 and 159 mg/dl**

|  | Comparing with Ezetimibe users | | | Comparing with Fenofibrates users | | | Comparing with Cholestyramine users | | |
| --- | --- | --- | --- | --- | --- | --- | --- | --- | --- |
| **Outcomes** | PCSK9 inhibitors cohort (%)^a^ | Ezetimibe cohort (%) | HR (95% CI)^a^ | PCSK9 inhibitors cohort (%)^a^ | Fenofibrates cohort (%) | HR (95% CI)^a^ | PCSK9 inhibitors cohort (%)^a^ | Cholestyramine cohort (%) | HR (95% CI)^a^ |
| All-caused dementia | 156 (1.0) | 303 (2.0) | 0.556 (0.458,0.675) | 108 (0.8) | 378 (2.9) | 0.348 (0.281,0.432) | 51 (0.9) | 155 (2.7) | 0.407 (0.297,0.56) |
| Vascular dementia | 38 (0.2) | 64 (0.4) | 0.643 (0.43,0.96) | 22 (0.2) | 94 (0.7) | 0.29 (0.182,0.462) | NA | NA | NA |
| Parkinson’s disease | 58 (0.4) | 62 (0.4) | 1.023 (0.715,1.464) | 40 (0.3) | 72 (0.6) | 0.673 (0.457,0.992) | 14 (0.2) | 33 (0.6) | 0.533 (0.285,1) |
| Alzheimer’s disease | 43 (0.3) | 81 (0.5) | 0.588 (0.406,0.851) | 28 (0.2) | 114 (0.9) | 0.307 (0.203,0.464) | 14 (0.2) | 47 (0.8) | 0.384 (0.211,0.698) |
| Other degenerative diseases of nervous system | 269 (1.7) | 311 (2.0) | 0.936 (0.795,1.102) | 195 (1.5) | 343 (2.6) | 0.698 (0.585,0.833) | 90 (1.6) | 182 (3.2) | 0.608 (0.472,0.784) |

PCSK9 inhibitors, Proprotein Convertase Subtilisin/Kexin Type 9 Inhibitor; In the TriNetX analytics platform, to maintain patient deidentification, incident case counts of 10 or fewer are censored and displayed as 10. Propensity score matching was implemented while controlling for baseline demographic variables (index age, sex, and race), body mass index, socioeconomic and lifestyle characteristics (socioeconomic or psychosocial–related health hazards, Family history of other mental and behavioral disorders (Z81.8), and mental or behavioral disorders associated with psychoactive substance use), healthcare utilization patterns (ambulatory and inpatient encounters), and a set of prespecified comorbidities, including essential hypertension, diabetes mellitus chronic ischemic heart disease, cerebral infarction, chronic kidney disease, and vitamin D deficiency.

**Table S32. Stratification analysis: population with HDL-C less than 39 mg/dl**

|  | Comparing with Ezetimibe users | | | Comparing with Fenofibrates users | | | Comparing with Cholestyramine users | | |
| --- | --- | --- | --- | --- | --- | --- | --- | --- | --- |
| **Outcomes** | PCSK9 inhibitors cohort (%)^a^ | Ezetimibe cohort (%) | HR (95% CI)^a^ | PCSK9 inhibitors cohort (%)^a^ | Fenofibrates cohort (%) | HR (95% CI)^a^ | PCSK9 inhibitors cohort (%)^a^ | Cholestyramine cohort (%) | HR (95% CI)^a^ |
| All-caused dementia | 152 (1.0) | 302 (2.1) | 0.548 (0.451,0.666) | 186 (1.1) | 585 (3.4) | 0.376 (0.319,0.444) | 97 (1.3) | 316 (4.2) | 0.357 (0.284,0.448) |
| Vascular dementia | 48 (0.3) | 79 (0.5) | 0.671 (0.469,0.961) | 64 (0.4) | 139 (0.8) | 0.561 (0.417,0.755) | 32 (0.4) | 72 (0.9) | 0.527 (0.347,0.8) |
| Parkinson’s disease | 56 (0.4) | 73 (0.5) | 0.844 (0.596,1.196) | 72 (0.4) | 118 (0.7) | 0.75 (0.559,1.008) | 32 (0.4) | 69 (0.9) | 0.537 (0.353,0.818) |
| Alzheimer’s disease | 37 (0.3) | 73 (0.5) | 0.565 (0.38,0.839) | 41 (0.2) | 157 (0.9) | 0.318 (0.225,0.449) | 22 (0.3) | 60 (0.8) | 0.434 (0.266,0.708) |
| Other degenerative diseases of nervous system | 213 (1.5) | 296 (2.0) | 0.785 (0.658,0.936) | 258 (1.5) | 468 (2.7) | 0.668 (0.573,0.778) | 128 (1.7) | 260 (3.4) | 0.578 (0.467,0.714) |

PCSK9 inhibitors, Proprotein Convertase Subtilisin/Kexin Type 9 Inhibitor; In the TriNetX analytics platform, to maintain patient deidentification, incident case counts of 10 or fewer are censored and displayed as 10. Propensity score matching was implemented while controlling for baseline demographic variables (index age, sex, and race), body mass index, socioeconomic and lifestyle characteristics (socioeconomic or psychosocial–related health hazards, Family history of other mental and behavioral disorders (Z81.8), and mental or behavioral disorders associated with psychoactive substance use), healthcare utilization patterns (ambulatory and inpatient encounters), and a set of prespecified comorbidities, including essential hypertension, diabetes mellitus chronic ischemic heart disease, cerebral infarction, chronic kidney disease, and vitamin D deficiency.

**Table S33. Stratification analysis: population with HDL-C less between 40 and 59 mg/dl**

|  | Comparing with Ezetimibe users | | | Comparing with Fenofibrates users | | | Comparing with Cholestyramine users | | |
| --- | --- | --- | --- | --- | --- | --- | --- | --- | --- |
| **Outcomes** | PCSK9 inhibitors cohort (%)^a^ | Ezetimibe cohort (%) | HR (95% CI)^a^ | PCSK9 inhibitors cohort (%)^a^ | Fenofibrates cohort (%) | HR (95% CI)^a^ | PCSK9 inhibitors cohort (%)^a^ | Cholestyramine cohort (%) | HR (95% CI)^a^ |
| All-caused dementia | 268 (1.1) | 426 (1.8) | 0.705 (0.605,0.821) | 238 (1.0) | 763 (3.1) | 0.376 (0.325,0.436) | 146 (1.2) | 460 (3.7) | 0.376 (0.312,0.453) |
| Vascular dementia | 65 (0.3) | 68 (0.3) | 0.829 (0.601,1.143) | 64 (0.3) | 175 (0.7) | 0.456 (0.342,0.608) | 42 (0.3) | 87 (0.7) | 0.589 (0.407,0.852) |
| Parkinson’s disease | 100 (0.4) | 112 (0.5) | 1 (0.764,1.31) | 98 (0.4) | 173 (0.7) | 0.689 (0.537,0.884) | 41 (0.3) | 107 (0.9) | 0.463 (0.323,0.665) |
| Alzheimer’s disease | 70 (0.3) | 112 (0.5) | 0.715 (0.53,0.964) | 57 (0.2) | 223 (0.9) | 0.317 (0.236,0.424) | 38 (0.3) | 110 (0.9) | 0.418 (0.289,0.606) |
| Other degenerative diseases of nervous system | 410 (1.7) | 487 (2.0) | 0.935 (0.82,1.067) | 399 (1.6) | 683 (2.8) | 0.714 (0.631,0.809) | 219 (1.8) | 432 (3.5) | 0.6 (0.51,0.707) |

PCSK9 inhibitors, Proprotein Convertase Subtilisin/Kexin Type 9 Inhibitor; In the TriNetX analytics platform, to maintain patient deidentification, incident case counts of 10 or fewer are censored and displayed as 10. Propensity score matching was implemented while controlling for baseline demographic variables (index age, sex, and race), body mass index, socioeconomic and lifestyle characteristics (socioeconomic or psychosocial–related health hazards, Family history of other mental and behavioral disorders (Z81.8), and mental or behavioral disorders associated with psychoactive substance use), healthcare utilization patterns (ambulatory and inpatient encounters), and a set of prespecified comorbidities, including essential hypertension, diabetes mellitus chronic ischemic heart disease, cerebral infarction, chronic kidney disease, and vitamin D deficiency.

**Table S34. Stratification analysis: population with triglyceride greater than 500 mg/dl**

|  | Comparing with Ezetimibe users | | | Comparing with Fenofibrates users | | | Comparing with Cholestyramine users | | |
| --- | --- | --- | --- | --- | --- | --- | --- | --- | --- |
| **Outcomes** | PCSK9 inhibitors cohort (%)^a^ | Ezetimibe cohort (%) | HR (95% CI)^a^ | PCSK9 inhibitors cohort (%)^a^ | Fenofibrates cohort (%) | HR (95% CI)^a^ | PCSK9 inhibitors cohort (%)^a^ | Cholestyramine cohort (%) | HR (95% CI)^a^ |
| All-caused dementia | NA | NA | NA | 22 (0.9) | 51 (2.2) | 0.525 (0.318,0.868) | NA | NA | NA |
| Vascular dementia | NA | NA | NA | NA | NA | NA | NA | NA | NA |
| Parkinson’s disease | NA | NA | NA | NA | NA | NA | NA | NA | NA |
| Alzheimer’s disease | NA | NA | NA | NA | NA | NA | NA | NA | NA |
| Other degenerative diseases of nervous system | NA | NA | NA | 35 (1.5) | 58 (2.5) | 0.714 (0.469,1.088) | 16 (1.7) | 22 (2.3) | 0.858 (0.45,1.638) |

PCSK9 inhibitors, Proprotein Convertase Subtilisin/Kexin Type 9 Inhibitor; In the TriNetX analytics platform, to maintain patient deidentification, incident case counts of 10 or fewer are censored and displayed as 10. Propensity score matching was implemented while controlling for baseline demographic variables (index age, sex, and race), body mass index, socioeconomic and lifestyle characteristics (socioeconomic or psychosocial–related health hazards, Family history of other mental and behavioral disorders (Z81.8), and mental or behavioral disorders associated with psychoactive substance use), healthcare utilization patterns (ambulatory and inpatient encounters), and a set of prespecified comorbidities, including essential hypertension, diabetes mellitus chronic ischemic heart disease, cerebral infarction, chronic kidney disease, and vitamin D deficiency.

**Table S35. Stratification analysis: population with triglyceride between 200 and 499 mg/dl**

|  | Comparing with Ezetimibe users | | | Comparing with Fenofibrates users | | | Comparing with Cholestyramine users | | |
| --- | --- | --- | --- | --- | --- | --- | --- | --- | --- |
| **Outcomes** | PCSK9 inhibitors cohort (%)^a^ | Ezetimibe cohort (%) | HR (95% CI)^a^ | PCSK9 inhibitors cohort (%)^a^ | Fenofibrates cohort (%) | HR (95% CI)^a^ | PCSK9 inhibitors cohort (%)^a^ | Cholestyramine cohort (%) | HR (95% CI)^a^ |
| All-caused dementia | 75 (1.3) | 110 (2.0) | 0.72 (0.537,0.966) | 190 (1.1) | 521 (3.0) | 0.438 (0.37,0.517) | 69 (0.9) | 257 (3.4) | 0.319 (0.244,0.416) |
| Vascular dementia | 20 (0.4) | 20 (0.4) | 1.051 (0.565,1.954) | 58 (0.3) | 114 (0.7) | 0.624 (0.454,0.858) | 20 (0.3) | 55 (0.7) | 0.457 (0.273,0.763) |
| Parkinson’s disease | 18 (0.3) | 28 (0.5) | 0.673 (0.372,1.216) | 73 (0.4) | 103 (0.6) | 0.866 (0.641,1.17) | 33 (0.4) | 77 (1.0) | 0.511 (0.339,0.769) |
| Alzheimer’s disease | 21 (0.4) | 33 (0.6) | 0.694 (0.401,1.199) | 50 (0.3) | 155 (0.9) | 0.395 (0.287,0.544) | 19 (0.3) | 59 (0.8) | 0.395 (0.235,0.663) |
| Other degenerative diseases of nervous system | 106 (1.9) | 113 (2.0) | 0.992 (0.761,1.293) | 301 (1.7) | 464 (2.7) | 0.787 (0.68,0.911) | 126 (1.7) | 254 (3.4) | 0.59 (0.476,0.731) |

PCSK9 inhibitors, Proprotein Convertase Subtilisin/Kexin Type 9 Inhibitor; In the TriNetX analytics platform, to maintain patient deidentification, incident case counts of 10 or fewer are censored and displayed as 10. Propensity score matching was implemented while controlling for baseline demographic variables (index age, sex, and race), body mass index, socioeconomic and lifestyle characteristics (socioeconomic or psychosocial–related health hazards, Family history of other mental and behavioral disorders (Z81.8), and mental or behavioral disorders associated with psychoactive substance use), healthcare utilization patterns (ambulatory and inpatient encounters), and a set of prespecified comorbidities, including essential hypertension, diabetes mellitus chronic ischemic heart disease, cerebral infarction, chronic kidney disease, and vitamin D deficiency.

**Table S36. Stratification analysis: population with total cholesterol greater than 240 mg/dl**

|  | Comparing with Ezetimibe users | | | Comparing with Fenofibrates users | | | Comparing with Cholestyramine users | | |
| --- | --- | --- | --- | --- | --- | --- | --- | --- | --- |
| **Outcomes** | PCSK9 inhibitors cohort (%)^a^ | Ezetimibe cohort (%) | HR (95% CI)^a^ | PCSK9 inhibitors cohort (%)^a^ | Fenofibrates cohort (%) | HR (95% CI)^a^ | PCSK9 inhibitors cohort (%)^a^ | Cholestyramine cohort (%) | HR (95% CI)^a^ |
| All-caused dementia | 146 (1.1) | 242 (1.7) | 0.652 (0.531,0.801) | 121 (0.9) | 363 (2.7) | 0.398 (0.323,0.489) | 47 (0.9) | 147 (2.9) | 0.377 (0.272,0.525) |
| Vascular dementia | 40 (0.3) | 65 (0.5) | 0.668 (0.45,0.991) | 30 (0.2) | 89 (0.7) | 0.413 (0.273,0.626) | NA | NA | NA |
| Parkinson’s disease | 37 (0.3) | 59 (0.4) | 0.692 (0.459,1.045) | 38 (0.3) | 56 (0.4) | 0.813 (0.537,1.229) | 16 (0.3) | 27 (0.5) | 0.716 (0.385,1.332) |
| Alzheimer’s disease | 39 (0.3) | 74 (0.5) | 0.581 (0.394,0.857) | 31 (0.2) | 96 (0.7) | 0.398 (0.265,0.598) | 11 (0.2) | 42 (0.8) | 0.31 (0.159,0.603) |
| Other degenerative diseases of nervous system | 219 (1.6) | 268 (1.9) | 0.89 (0.744,1.064) | 187 (1.4) | 326 (2.4) | 0.69 (0.576,0.827) | 66 (1.3) | 151 (3.0) | 0.512 (0.383,0.685) |

PCSK9 inhibitors, Proprotein Convertase Subtilisin/Kexin Type 9 Inhibitor; In the TriNetX analytics platform, to maintain patient deidentification, incident case counts of 10 or fewer are censored and displayed as 10. Propensity score matching was implemented while controlling for baseline demographic variables (index age, sex, and race), body mass index, socioeconomic and lifestyle characteristics (socioeconomic or psychosocial–related health hazards, Family history of other mental and behavioral disorders (Z81.8), and mental or behavioral disorders associated with psychoactive substance use), healthcare utilization patterns (ambulatory and inpatient encounters), and a set of prespecified comorbidities, including essential hypertension, diabetes mellitus chronic ischemic heart disease, cerebral infarction, chronic kidney disease, and vitamin D deficiency.

**Table S37. Stratification analysis: population with total cholesterol between 200 and 239 mg/dl**

|  | Comparing with Ezetimibe users | | | Comparing with Fenofibrates users | | | Comparing with Cholestyramine users | | |
| --- | --- | --- | --- | --- | --- | --- | --- | --- | --- |
| **Outcomes** | PCSK9 inhibitors cohort (%)^a^ | Ezetimibe cohort (%) | HR (95% CI)^a^ | PCSK9 inhibitors cohort (%)^a^ | Fenofibrates cohort (%) | HR (95% CI)^a^ | PCSK9 inhibitors cohort (%)^a^ | Cholestyramine cohort (%) | HR (95% CI)^a^ |
| All-caused dementia | 199 (1.1) | 312 (1.7) | 0.697 (0.584,0.833) | 157 (1.0) | 476 (2.9) | 0.409 (0.342,0.491) | 91 (1.1) | 253 (3.2) | 0.45 (0.354,0.572) |
| Vascular dementia | 46 (0.3) | 62 (0.3) | 0.81 (0.553,1.187) | 35 (0.2) | 115 (0.7) | 0.391 (0.267,0.571) | 25 (0.3) | 60 (0.8) | 0.539 (0.337,0.861) |
| Parkinson’s disease | 67 (0.4) | 91 (0.5) | 0.808 (0.589,1.109) | 53 (0.3) | 88 (0.5) | 0.757 (0.538,1.067) | 24 (0.3) | 54 (0.7) | 0.541 (0.334,0.877) |
| Alzheimer’s disease | 64 (0.4) | 95 (0.5) | 0.745 (0.543,1.024) | 52 (0.3) | 143 (0.9) | 0.455 (0.331,0.626) | 27 (0.3) | 74 (0.9) | 0.472 (0.303,0.735) |
| Other degenerative diseases of nervous system | 332 (1.9) | 363 (2.0) | 0.996 (0.858,1.156) | 262 (1.6) | 460 (2.8) | 0.708 (0.608,0.824) | 140 (1.8) | 264 (3.3) | 0.661 (0.538,0.813) |

PCSK9 inhibitors, Proprotein Convertase Subtilisin/Kexin Type 9 Inhibitor; In the TriNetX analytics platform, to maintain patient deidentification, incident case counts of 10 or fewer are censored and displayed as 10. Propensity score matching was implemented while controlling for baseline demographic variables (index age, sex, and race), body mass index, socioeconomic and lifestyle characteristics (socioeconomic or psychosocial–related health hazards, Family history of other mental and behavioral disorders (Z81.8), and mental or behavioral disorders associated with psychoactive substance use), healthcare utilization patterns (ambulatory and inpatient encounters), and a set of prespecified comorbidities, including essential hypertension, diabetes mellitus chronic ischemic heart disease, cerebral infarction, chronic kidney disease, and vitamin D deficiency.

**Supplementary figures**

**Figure S1.** Cumulative probability of all-caused dementia, vascular dementia, Parkinson’s disease, Alzheimer’s disease and other degenerative diseases of nervous system in PCSK9 inhibitors users and different active comparators (statin, ezetimibe, fenofibrates and cholestyramine)

*Legends:*

In each analysis presented in this figure, lagging period was set as 1 month after index date and follow-up period was set as 5 year. Propensity score matching was implemented while controlling for baseline demographic variables (index age, sex, and race), body mass index, socioeconomic and lifestyle characteristics (socioeconomic or psychosocial–related health hazards, Family history of other mental and behavioral disorders (Z81.8), and mental or behavioral disorders associated with psychoactive substance use), healthcare utilization patterns (ambulatory and inpatient encounters), and a set of prespecified comorbidities, including essential hypertension, diabetes mellitus chronic ischemic heart disease, cerebral infarction, chronic kidney disease, and vitamin D deficiency.
